# Value-Based Healthcare From the Perspective of the Healthcare Professional: A Systematic Literature Review

**DOI:** 10.3389/fpubh.2021.800702

**Published:** 2022-01-13

**Authors:** Veerle van Engen, Igna Bonfrer, Kees Ahaus, Martina Buljac-Samardzic

**Affiliations:** Erasmus School of Health Policy & Management, Erasmus University Rotterdam, Rotterdam, Netherlands

**Keywords:** value-based healthcare, VBHC, healthcare professional, job demands, job resources, employee well-being, literature review, psychosocial perspective

## Abstract

**Introduction:** Healthcare systems increasingly move toward “value-based healthcare” (VBHC), aiming to further improve quality and performance of care as well as the sustainable use of resources. Evidence about healthcare professionals' contributions to VBHC, experienced job demands and resources as well as employee well-being in VBHC is scattered. This systematic review synthesizes this evidence by exploring how VBHC relates to the healthcare professional, and vice versa.

**Method:** Seven databases were systematically searched for relevant studies. The search yielded 3,782 records, of which 45 were eligible for inclusion based on a two-step screening process using exclusion criteria performed by two authors independently. The quality of the included studies was appraised using the Mixed Methods Appraisal Tool (MMAT). Based on inductive thematic analysis, the Job Demands-Resources (JD-R) model was modified. Subsequently, this modified model was applied deductively for a second round of thematic analysis.

**Results:** Ten behaviors of healthcare professionals to enhance value in care were identified. These behaviors and associated changes in professionals' work content and work environment impacted the experienced job demands and resources and, in turn, employee well-being and job strain. This review revealed 16 constructs as job demand and/or job resource. Examples of these include role strain, workload and meaning in work. Four constructs related to employee well-being, including engagement and job satisfaction, and five constructs related to job strain, including exhaustion and concerns, were identified. A distinction was made between job demands and resources that were a pure characteristic of VBHC, and job demands and resources that resulted from environmental factors such as how care organizations shaped VBHC.

**Conclusion and Discussion:** This review shows that professionals experience substantial job demands and resources resulting from the move toward VBHC and their active role therein. Several job demands are triggered by an unsupportive organizational environment. Hence, increased organizational support may contribute to mitigating or avoiding adverse psychosocial factors and enhance positive psychosocial factors in a VBHC context. Further research to estimate the effects of VBHC on healthcare professionals is warranted.

## Introduction

Healthcare systems increasingly move toward “value-based healthcare” (VBHC) (1), aiming to further improve quality and performance of care as well as the equitable, sustainable, and transparent use of resources (1–3). Thus far, a globally shared definition of VBHC is lacking (4). Yet, a characteristic shared by most VBHC programs is the multifaceted approach that, on top of clinical outcomes, provides a prominent place to patient-reported quality and performance indicators. Examples of these include “Patient Reported Outcome Measures” (PROMs) and “Patient Reported Experience Measures” (PREMs) (2).

The early initiators of VBHC state that, in addition to improving health value, employee well-being should be part of healthcare organizations' imperatives since healthcare professionals play a central role in VBHC (1). This aligns to the quadruple aim of (1) improving health outcomes for patients, (2) enhancing patient experience, (3) enhancing healthcare professional experience, and (4) reducing cost (5). In comparison to traditional care practices, VBHC may change professionals' work by introducing new, or shifting emphasis toward, value-promoting care activities and team-based care (6). Such activities include discussing value with patients, making a shared decision, learning, and improving based on quality and performance indicators and providing care in pathways (7–9). Although these activities may not all be completely new (10), the difference is that each activity is now used as a means to generate value rather than being an end-goal in itself. VBHC is different from current care and requires new competencies of professionals (11). Psychosocial factors at work describe how work factors, such as the work environment and job content, interact with personal factors, such as a person's competence and expectations, to impact employee experience and well-being (12, 13). Hence, we may expect changes in professionals' well-being with VBHC currently gaining traction.

However, to date, evidence from studies taking a psychosocial perspective on VBHC, with insights about how professionals contribute to VBHC and how VBHC influences their well-being, is scattered. Most studies on VBHC understandably focus on patients and clinical results (14–16) and build on insights from implementation science [e.g., (17–19)]. Earlier reviews focusing on healthcare professionals and VBHC studied education (20) and interventions to reduce low-value behavior (21). Current literature suggests that VBHC meets the interest of professionals i.e., to deliver value for patients (1) and positively contributes to their work experience (22). However, the relation between VBHC and professionals' interests nor the contribution of VBHC to their work experience has been convincingly established. Current literature hints at a relation between VBHC and various job demands and resources including work pressure, emotional demands, and autonomy (23). The literature further suggests both positive and negative relations between VBHC and professionals' well-being, such as improved engagement (24) and potential fears concerning among others accountability and value-based competition on results (1).

This systematic literature review synthesizes empirical findings centering around the question “how does VBHC relate to the healthcare professional and vice versa?”. The review aims to provide a comprehensive overview of professionals' roles in VBHC, experienced job demands and resources as well as the impact that value-based work can have on professionals' well-being. This work may contribute to mitigating or avoiding adverse psychosocial factors at work for healthcare professionals in VBHC and enhance positive psychosocial factors.

## Methods

This systematic review followed the PRISMA2020 guidelines (Preferred Reporting Items for Systematic Reviews and Meta-Analyses) (25).

### Search Strategy

An extensive three-armed search strategy was developed in consultation with the Erasmus Medical Center's Medical Library. The search string followed the PICO statement by including keywords that describe (1) the *population*, i.e., healthcare professionals, their teams or specific occupations, (2) the *intervention*, i.e., VBHC, and (3) *outcomes*, i.e., how the population impacts VBHC or vice versa (see Supplementary Material 1). The *comparator* is not applicable in this work.

The first part of the search string included generic descriptions of professionals or care teams, such as “professional,” “staff,” “nurse,” and “clinician,” as well as specific occupations derived from the International Standard Classification of Occupations ISCO-08 (26). Occupations both in hospital and other healthcare settings were included.

In line with terminology used by Porter and Teisberg (1), we included “high-value care” and “value driven care” in the search string as synonyms for VBHC. In the second arm of the search strategy, we searched for the use of “value-based” OR “valuebased” OR “high-value” OR “value-driven” mentioned within three words-distance of the word “care” OR “healthcare” since a Medical Subject Heading (MeSH) term for VBHC is missing. Studies only reporting on value-based payment methods were excluded, as these are beyond the scope of our work.

Third, we searched for keywords describing a relation, a characteristic or action of a professional or an outcome relevant to professionals. Examples of keywords describing a relation were “affect,” “cause,” and “benefit.” Keywords describing a characteristic or action of a professional included, among others, “attitude,” “knowledge,” and “behavior.” Keywords describing an outcome relevant to professionals were abstracted from relevant literature and lists of human values (27, 28) and included, among others, “workload,” “autonomy,” and “engagement.”

The search string was piloted by checking whether a pre-selected set of 10 relevant studies was indeed retrieved when conducting the search, which was the case for all 10 studies. Supplementary Material 1 contains the full search string and further explanation. The search was performed on December 21, 2020 in seven databases, being Embase.com, Medline ALL Ovid., PsycINFO ALL Ovid, Web of Science (SCI-EXPANDED & SSCI), CINAHL EBSCOhost, Business Source Premier EBSCOhost and EconLit ProQuest. Conference papers were excluded.

### Selection Process

A two-step screening process, comprising title and abstract screening and full-text assessment, was performed by two of the authors independently. Titles and abstracts screening resulted in eligible studies for full-text assessment. In both steps, studies were subjected to pre-defined eligibility criteria. Papers with inconsistent screening outcomes between the first- and second-screener during title and abstract screening were included for full-text assessment. In case of inconsistent screening outcomes in full-text assessment, authors discussed the paper and when no consensus was reached full-text assessment by the last author was decisive. This was the case for three papers.

### Eligibility Criteria

The exclusion criteria for all yielded studies were “not a peer-reviewed paper and/or journal,” “no empirical data,” “not part of/contributing to VBHC or synonym,” “no relation to the healthcare professional,” “only about VBHC education,” “only about value-based payment or synonym,” and “non-English.” In absence of consensus on a VBHC definition (4), we relied on the authors' judgement i.e., any study in which the original author identified the intervention as “value-based healthcare” or its synonyms was assumed to be about VBHC. We identified a healthcare professional as anyone caring for, or aiming to cure, patients or clients with a formal training to do so. Consequently, consultants, administrative staff and data analyst, among others, were not considered as healthcare professionals.

### Data Extraction

Data extraction comprised two steps. First, general study characteristics were extracted. This was followed by data extraction on the relation between VBHC and the healthcare professional.

#### General Study Characteristics

Elements for generic data extraction were informed by discussion among all authors and included year of publication, country, study aim, study design, healthcare setting, profession, healthcare discipline, VBHC terminology, VBHC components applied, and the degree of professionals' involvement in VBHC. Data were abstracted by the first author.

#### The Relation Between VBHC and the Healthcare Professional

First, an inductive approach was applied to analyze the relation between VBHC and the healthcare professional using thematic analysis (28). This started with familiarization with the “Results” sections in the included studies and selection of relevant quotes. Afterwards, semantic codes that closely reflected the original authors wording were attached to the selected quotes. Subsequently, repeated patterns of meaning in these codes were clustered to generate latent themes describing the underlying codes. Last, the themes were revised and possible interconnectivity between themes was indicated to derive a thematic map. Atlas.TI software was used to facilitate this process.

The resulting thematic map showed various similarities with the Job Demands-Resources (JD-R) conceptual model (12). JD-R is a recognized psychosocial model applied to explore and design the interaction between “the job” and “the professional”. More specifically, JD-R describes that work has certain characteristics that make professionals feel engaged or strained, depending on whether these are perceived to give energy, i.e., job resources, or take energy, i.e., job demands. The level of engagement and job strain can subsequently be used to predict performance. Since JD-R allows flexible use and tailoring to fit specific contexts (29), we iteratively adapted the JD-R model by including all abstracted data regarding the relation between VBHC and the professional. Use of JD-R as an underlying conceptual model allowed for our findings to be compared to earlier scholarly work on job demands and resources.

Subsequently, the resulting modified JD-R model was used for deductive analysis. Quotes from the “Results” sections in the included studies were selected and attached to one or multiple components of the modified JD-R model using Atlas.TI software. Consistent with the eligibility criteria, data about value-based payment and VBHC education were omitted. The resulting quotes were analyzed at both a latent and semantic level. The latent approach was applied to define whether experiences were a job resource or demand as this was often not explicitly mentioned. Next, we worked from the wording as used by the original author to inductively cluster similar data within the JD-R components to form codes. The resulting codes included among others “workload” and “joy in practice.” Overall, the analysis process was iterative and evolved from description to interpretation. Throughout this process the descriptive evidence and interpretations were discussed with all co-authors to validate line of reasoning, comprehensiveness and adequate representation of the included studies.

### Quality Appraisal

Quality appraisal of the included studies was performed using the Mixed Methods Appraisal Tool (MMAT) (30), which is applicable to qualitative, quantitative, and mixed methods studies. For each study design, MMAT provides a set of five quality criteria. Mixed methods studies were assessed on both the qualitative and quantitative set of criteria and a complementary set that specifically appraises the quality of the mixed methods design. The scores resulted in a classification of each study into “high,” “medium,” or “low” research quality. Supplementary Material 2 provides details on the scoring methodology and MMAT scores for each included study. Quality appraisal was used to provide an overall impression of the study quality. No studies were excluded based on the MMAT scores.

## Results

### Selected Studies

**Figure 1:**
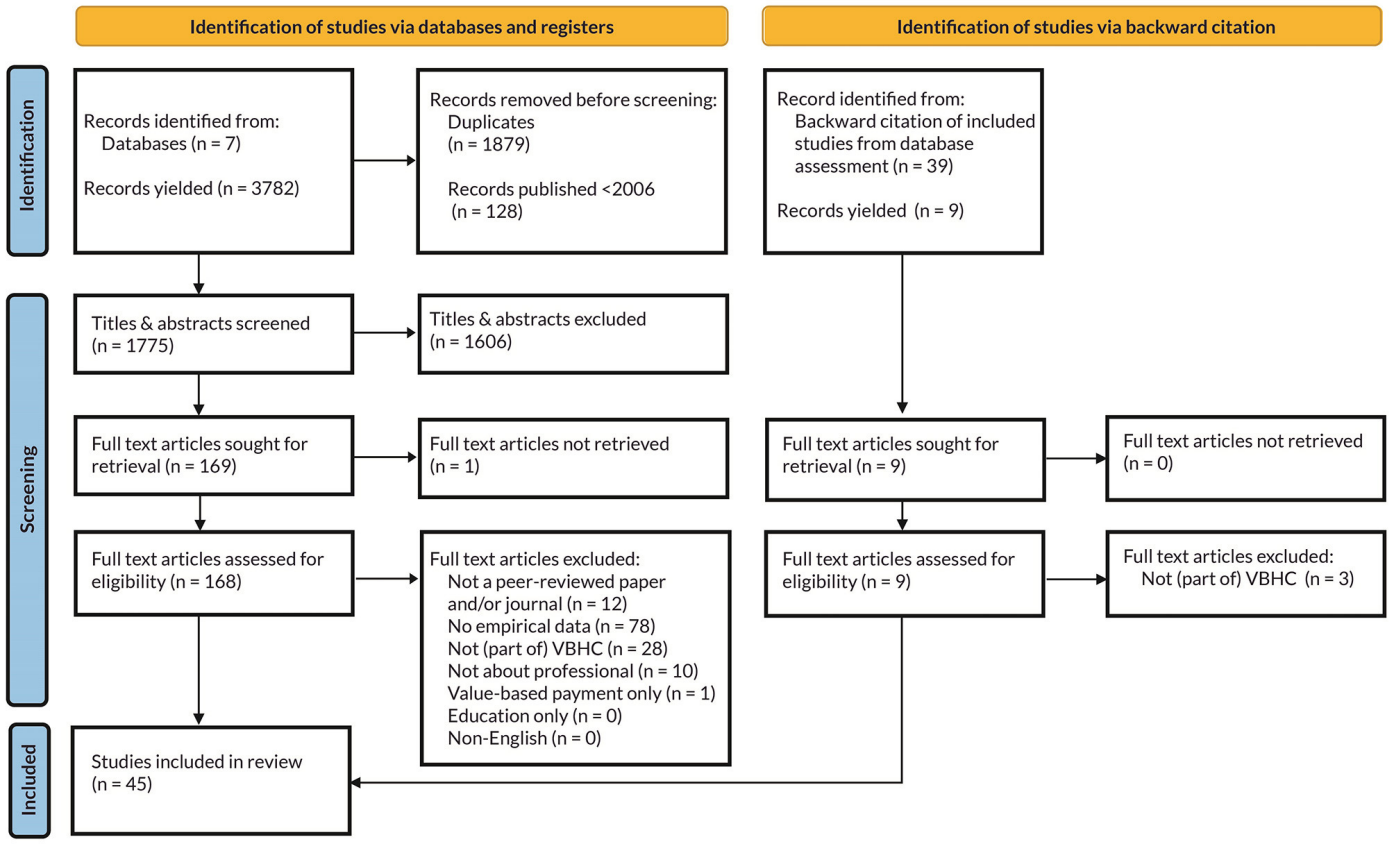
The PRISMA flow diagram following PRISMA2020 guidelines (25).

The search yielded 3,782 records. Duplicates and literature published earlier than the introduction of VBHC in 2006 (1) were removed, resulting in 1,775 papers for title and abstract screening. Finally, backward citation searching of the included studies resulted in inclusion of six additional papers. Based on the assessment using the exclusion criteria, 45 studies were eligible for inclusion. Figure 1 displays the corresponding PRISMA diagram.

#### Study Characteristics

Of the 45 included studies, 23 had a qualitative study design, 14 were quantitative and 8 applied mixed methods. Supplementary Material 3 contains the full list of included studies and a summary table.

#### Study Setting

Healthcare professionals from the USA (*n* = 23), Sweden (*n* = 8), and The Netherlands (*n* = 7) were most frequently studied. No studies were performed in low-income countries. Four Swedish studies reported on the same intervention and population (24, 31–33). Hence, from the 45 studies included in this review 42 are unique.

From all studies, 24 took place in a hospital. The other studies focused on “accountable care organizations” (ACOs) (*n* = 2), primary care (*n* = 2), ambulatory care (*n* = 2), medical laboratory (*n* = 1), oral healthcare (*n* = 1), home care (*n* = 1), not applicable/specified (*n* = 3), or different combinations of care settings (*n* = 9), which included the above and new settings such as elderly care, maternity care, midwifery practice, and physiotherapy. The included studies focused on various medical specialties such as internal medicine, orthopedics and cardiovascular care. The studied populations were trained healthcare professionals (*n* = 31), residents (*n* = 7), or a combination of both (*n* = 2). Five studies focused on other healthcare actors or did not specify the composition of professionals involved.

#### Defining VBHC

“Value-based healthcare” (VBHC) has been used as term by 27 studies, followed by “high-value care” (HVC) (*n* = 12) and “high-value, cost-conscious care” (HVCCC) (*n* = 4). Two studies used terms interchangeably. For the readability of this review, the term VBHC will be used in the remainder of this text to refer to all of the previous.

VBHC in general, without specification of the value-enhancing interventions, was studied in 11 studies. The other studies primarily reported on team-based care models, outcome measures, quality improvement, discussing value in the clinical encounter, cost-consciousness, and care coordination within the organization's walls as specific components of VBHC. Less frequently studied VBHC components included population health, prevention, collaboration in the full care chain and redesign of pathways and workflows. In 24 studies the population actively participated in a VBHC intervention. In 19 studies it was uncertain to what degree participants were involved in VBHC, for example studies evaluating VBHC awareness and beliefs. Two studies did not collect data directly from professionals. These studies focused on open workforce positions in VBHC and development of a framework regarding professionals' roles in VBHC.

#### Research Design and Quality

Whereas few studies explicitly investigated the implementation process of VBHC [e.g., (24, 34, 35)], the majority of studies did not clarify the time frame between VBHC implementation and data collection for scholarly work. Other than one study deploying the JD-R model (23), none of the included studies built on existing conceptual models. Five validated research instruments to study VBHC in relation to the healthcare professionals were used, containing three full-scales (36–38), one sub-scale (39), and one observer-based instrument (40).

Quality appraisal showed that 22 studies were rated as high quality, 12 studies medium quality, and 11 studies low quality. Supplementary Material 2 provides details. Overall, qualitative studies scored highest and mixed methods studies had the lowest scores.

### The Modified JD-R Model

**Figure 2:**
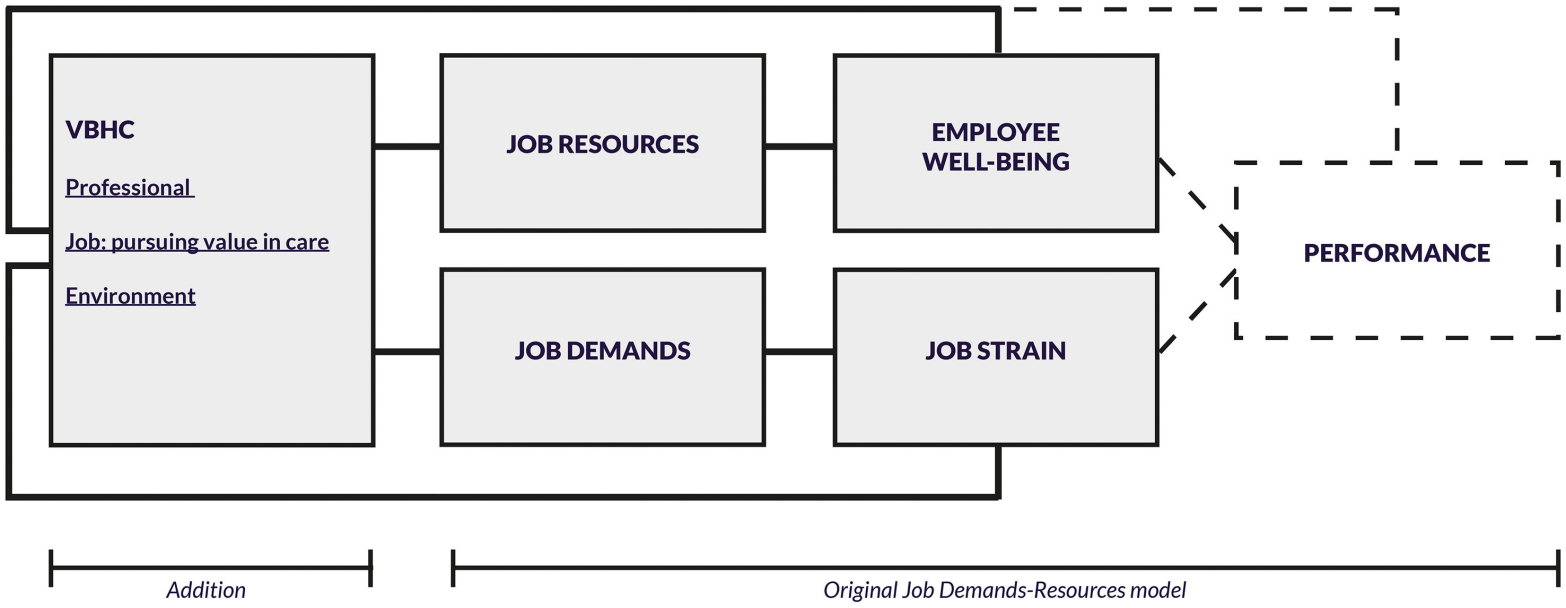
The modified JD-R model that was informed by inductive analysis and subsequently used for deductive analysis.

Figure 2 presents the modified JD-R model that the authors developed based on inductive analysis, subsequently applied for deductive analysis. Two modifications were made to the original JD-R model (12). First, an additional column was added on the left-side with elements specific to VBHC. These included the “professional,” the “job” of pursuing value in care and the “environment” in which VBHC takes place. This additional column allowed studying antecedents of job demands and resources. The column in the middle reflected the demands and resources that professionals experienced when providing VBHC. These demands and resources were connected to the right column comprising the constructs of employee well-being and job strain.

Second, as outcomes of employee well-being and job strain, we distinguished between “day-to-day” performance and long-term performance. The JD-R construct “performance” at the end of the conceptual model was omitted as it suggests a long-term focus. Although work can impact professionals' long-term performance, such as absence and intention to leave practice (41, 42), we concluded from the analysis of the included studies that VBHC needs to mature before it is possible to observe long-term effects of VBHC on professionals' performance. Hence, outcomes related to employee well-being and job strain were linked back to the left column that described the professionals' day-to-day performance in value-based work. Patient performance, such as health outcomes (18, 43), and organizational performance, such as operational and performance metrics (44, 45), have been studied. However, these were omitted as they are not the scope of this study.

### Thematic Analysis

**Figure 3:**
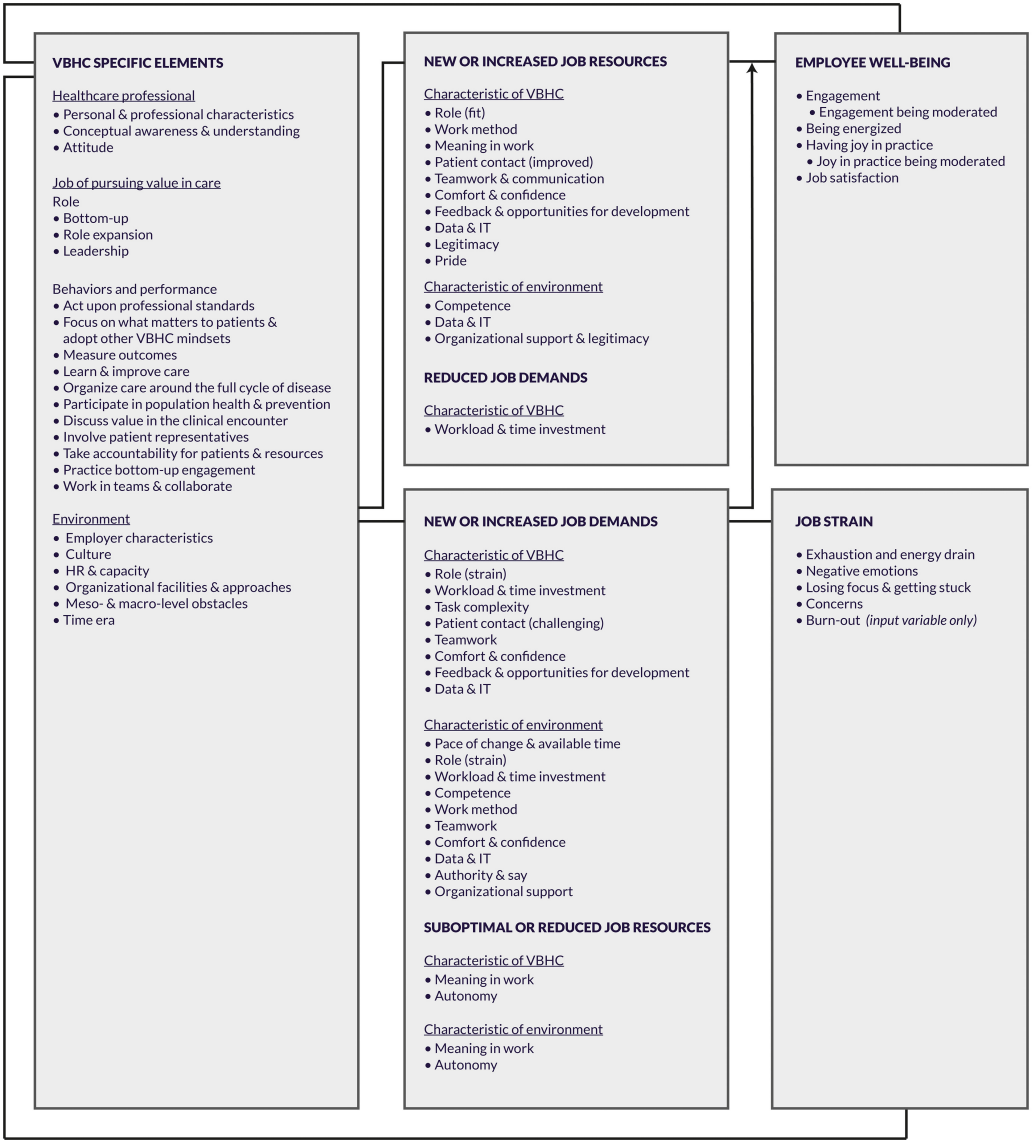
Psychosocial factors identified from thematic analysis using the modified JD-R model.

Over 800 quotes that resulted from the 45 included studies were thematically analyzed using the modified JD-R model. Figure 3 shows that VBHC was associated with specific job demands and resources. Besides providing an overview of these factors, we distinguished between two types of job demands and resources. Namely, job demands and resources that were purely informed by the characteristics of the job, in this case pursuing VBHC, and job demands and resources that stemmed from characteristics of the environment. These characteristics of the environment included among others organizational structures, culture, and resources, as well as how actors, such as healthcare organizations and policy makers, facilitated, and shaped the job. For example, when a professional experienced that VBHC took more effort than traditional care, this was considered a demand that resulted from the nature of VBHC. When a professional felt pressured by the pace of implementation, this was considered a demand triggered by a characteristic of the environment. Connecting lines in Figure 3 were based on the studies included in the review and hence differ from the original JD-R model. Except for an arrow describing the moderating effect that job demands may have on the relationship between job resources and employee well-being, arrows in the model were omitted to reflect possible bidirectionality.

#### Summary of the VBHC Specific Elements

For conciseness, the findings of the VBHC specific elements (left column in Figure 3) are summarized below. Details are provided in Supplementary Material 4. The VBHC specific elements comprised “the professional,” “the job,” and “the environment” as described from the professional's perspective.

##### The Professional

We identified three topics related to the healthcare professional, namely (1) personal and professional characteristics, (2) conceptual awareness and understanding, and (3) attitudes toward VBHC. Regarding “personal and professional characteristics” studies investigated, among others, age, job function, and professional values in relation to VBHC awareness (46, 47). Other studies showed mixed results regarding gender and job function in relation to VBHC attitudes and scores (23, 48, 49). Second, scholars investigated professionals' conceptual awareness (33, 46, 47, 50) and understanding (24, 31, 33, 35, 46, 49, 51–55) of VBHC, which revealed variation and possible prioritization of either patient outcomes or resource consciousness. Last, professionals' attitudes to VBHC were shown to be positive (14, 23, 24, 31, 33–35, 46, 48, 53–58) and/or negative (23, 24, 31, 33–35, 38, 47, 50–53, 55, 57–59). Positive attitudes included professionals mentioning that VBHC was received with hope (35), convincement (24), excitement and enthusiasm (33), and with suggested readiness (58). Negative attitudes included critique (53), perceived drawbacks (23) and resistance (24, 47, 51, 55), especially in the light of considering costs (38, 52, 55, 57) and discussing costs with patients (57, 58).

##### The Job of Pursuing Value in Care

**Figure 4:**
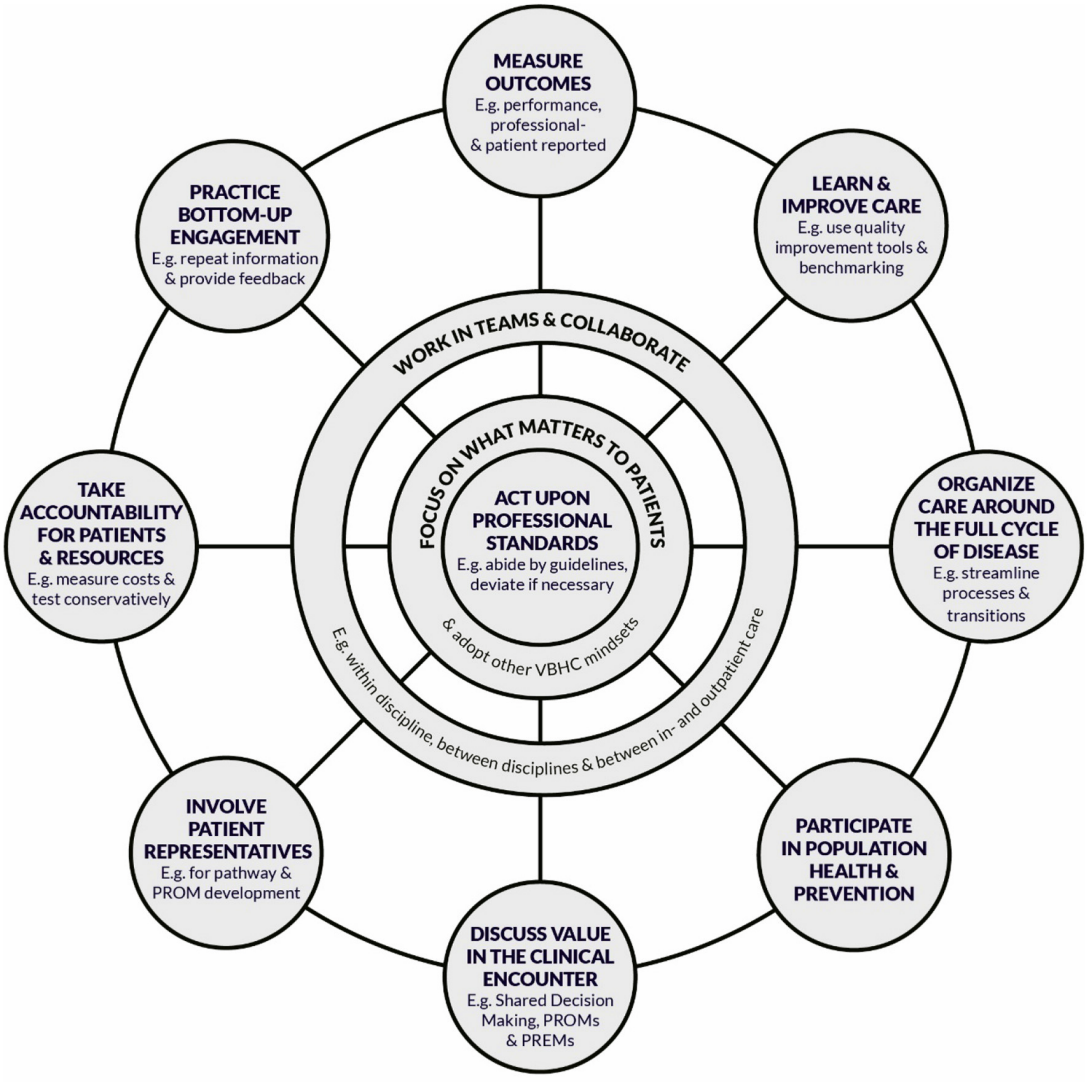
Professionals' behaviors to pursue value in care identified from thematic analysis.

Related to professionals' roles and behaviors, studies described VBHC as a bottom-up initiative (14, 24, 31, 32, 34, 47, 53, 54) that expanded roles and established new roles such as the “contact nurse” function (14, 24, 32, 56, 60–66). Engaged leadership was studied in terms of necessity, leadership approaches, competence, personal characteristics, as well as professions that were suggested to take up leadership roles (33, 34, 54, 66, 67). Analysis revealed 10 specific behaviors that professionals pursued in VBHC, next to acting upon their professional standards (68). These interconnected and mutually reinforcing behaviors, as visualized in Figure 4, are to (1) focus on what matters to patients and adopt other VBHC mindsets (24, 31–33, 47, 50, 52, 53, 61, 62), (2) measure outcomes (14, 24, 31–35, 44, 56, 68), (3) learn and improve care (14, 24, 31–34, 47, 53, 62, 66, 68–70), (4) organize care around the full cycle of disease (24, 32, 44, 45, 54, 60, 61, 64, 66, 70–73), (5) participate in population health and prevention (24, 62, 66, 70, 72), (6) discuss value in the clinical encounter (31, 47, 50, 55, 56, 58, 63, 64, 74, 75), (7) involve patient representatives (24, 31–33, 50), (8) take accountability for patients and resources (31, 33, 38, 44, 47, 48, 54–57, 60, 64, 68, 69, 74, 75), (9) practice bottom-up engagement (14, 24, 31–35, 47), and above all (10) work in teams and collaborate (31, 34, 50, 61, 62, 66, 68, 72, 76).

##### The Environment

Related to the perceived VBHC environment, six factors were identified, namely (1) employer characteristics, (2) culture, (3) Human Resources (HR) and capacity, (4) organizational facilities and approaches, (5) meso- and macro-level obstacles, and (6) the time era. First, studied employer characteristics included hospital type, region, health-care intensity, and number of clinicians. These factors were related to, among others, self-reported knowledge, perceived barriers, behaviors, and performance in VBHC (23, 48, 55, 63, 64, 69, 70, 74, 75, 77). Concerning culture, participants called for culture change (24, 31, 47, 56) and mentioned the need for specific cultures, particularly cultures that are transparent and blame-free (14, 31, 48, 53, 56, 66, 69, 72). Related to HR and capacity, studies discussed staffing constraints (33, 49, 59, 61), the importance of staff stability (24, 59, 66), staff composition including the use of alternative providers and medical assistants [e.g., (14, 24, 33, 35, 60, 64–66, 71, 72, 76)] and specific open job positions (33, 34, 62, 72, 78). Remarks made about organizational facilities and approaches involved professionals' desire for dedicated VBHC time (14, 59, 66), step-by-step implementation (34, 35, 56, 72, 76), and an overall supportive environment (24, 31, 53, 54, 56, 57, 59, 62, 65, 66, 72, 73) with specific attention for engaged leadership (14, 33, 35, 54, 66). Analysis revealed several meso- and macro-level impediments to VBHC (24, 35, 49, 54, 76) such as current waitlists to access specialists. Last, related to the time era, one study reported on the expected progressive impact of Covid-19 on VBHC (72).

#### Job Demands and Job Resources

One study specifically investigated job resources and job demands in relation to professionals' attitudes toward high-value care, cost incorporation and perceived drawbacks using JD-R (23). Associations, both positively and negatively, were found for the following job demands and resources: autonomy, work pressure, opportunities for development, supervisory coaching, cognitive demands, and emotional demands.

In combination with the remaining studies, thematic analysis identified 16 job demands and resources (see Figure 3), namely: role fit or role strain, workload and time investment, competence, task complexity, work method, meaning in work, improved or more challenging patient contact, teamwork and communication, comfort and confidence, feedback and opportunities for personal development, pace of change and time availability, data and IT, authority and say, autonomy, organizational support and legitimacy, and lastly, pride. Most of these constructs can both be a demand and resource depending on whether they give or take energy. All aforementioned constructs are discussed below. An overview is provided in Table 1.

**Table 1:** Overview and illustrative quotes on job demands and resources in VBHC.

**Job demands and Resources**	**Specification**	**Studies**	**Exemplifying quote**
Role (fit and strain)	VBHC resource	(24, 31, 35, 54, 55, 66, 72)	“*It seems that VBHC appeals to healthcare professionals' closest sphere of interest”* (31)
	VBHC demand	(14, 24, 32, 34, 51, 55, 60, 69, 74)	“*Another problem was that team leaders found it difficult to prioritize their implementation work because they felt that their patients were their first priority”* (33)
	Environment demand	(33, 38, 55)	“*Adding to the complexity of learning to provide HV3C were the mixed messages that residents received at the workplace level regarding their role in HV3C”* (55)
Workload and time investment	VBHC resource	(60, 66, 72)	“*[..] medical assistants would room patients, ensure all paperwork was printed and complete, and act as scribes entering most of the information into the EHR. This allowed physicians to focus on patients, not the HER [electronic health record]. As one physician stated, “I got to practice medicine again!”* (66)
	VBHC demand	(31–33, 51, 62, 66, 71, 73)	*[The most common barriers to high-value care Included:] “increased time and effort”* (51)
	Environment demand	(33)	“*However, the difficulties of accessing data, especially from the internal IT system, took too much time and energy because it required so much manual work”* (33)
Competence	Environment resource	(75, 77, 79)	“*The highest measured mean scores were found in the competence areas ‘Value-based nursing care’ and […]”* (79)
	Environment demand	(31, 33, 38, 49, 50, 55, 57, 59, 60, 62, 75)	“*Our experts did have the concern that […] many lack the skills and training to take advantage of those data, whether the data were ‘mined’ by themselves or by a data scientist”* (62)
Task complexity	VBHC demand	(60, 73)	“*Participants shared another disadvantage of CPW [clinical pathways] is ‘information overload,’ where the number and length of pathways are perceived to be increasing over time. Providers find it challenging to remain up-to-date on which pathways exist and are unable to educate oneself on the content”* (73)
Work method	VBHC resource	(24, 31, 32, 49, 50)	“*Participants stated that VBHC includes improved working methods and organization of the work”* (31)
	VBHC demand	(14, 49)	“*A systematic approach for the identification of improvement potential, and the selection and implementation of improvement initiatives is lacking. Physicians explicitly mentioned that they struggle with this.”* (14)
	Environment demand	(24)	“*This organizational structure was frustrating as this contributed to difficulties in tracking and following patients during the course of the disease when they crossed boundaries between departments”* (24)
Meaning in work	VBHC resource	(24, 31, 32, 35, 56, 60, 61, 66, 73)	“*The presence of medical assistants, care coordinators, and other team members, in conjunction with population management tools, created the opportunity to better understand, manage, and care for individual patients and different populations”* (66)
	VBHC demand	(24, 31)	“*Engagement for VBHC also decreased when participants did not see any actual activity or result of their implementation work”* (24)
	Environment demand	(24)	“*Being forced to make cancellations caused frustration among participants. They then lost their confidence in working with VBHC and found it meaningless trying to make smaller changes in the process when the great problem was lack of capacity”* (24)
Patient contact (improved and challenging)	VBHC resource	(50, 61, 73)	“*CPW [clinical pathways] not only improve communication among team members but facilitate conversations with patients and families regarding plans of care”* (73)
	VBHC demand	(49–51, 55, 57, 69, 73–75)	“*Nearly 40% reported that clinicians are uncomfortable discussing the costs of tests or treatments with patients and reported that clinicians do not feel that physicians should discuss costs with patients”* (57)
Teamwork and communication	VBHC resource	(24, 44, 53, 61, 66, 73)	“*Planning the production also included improvements in the communication between in- and outpatient wards”* (32)
	VBHC demand	(24)	“*People get confused when we have to start working between silos according to the principle of value for the patients”* (24)
	Environment demand	(33, 47, 49, 51, 73)	“*This pressure to comply results in providers describing feelings of guilt when non-adherent, which can prevent high-quality care and create conflict within a team”* (73)
Comfort and confidence	VBHC resource	(73)	“*CPW [clinical pathways] offer the additional benefit of providing practice validation, fostering confidence, and affirming clinical decision-making skills”* (73)
	VBHC demand	(48, 51, 55, 60, 69, 75)	*[Certified Medical Assistants mention] “a lack of comfort with the complexity of the new tasks”* (60)
	Environment demand	(33)	“*The participants were also uncertain as to whether or not this manual work could negatively influence the validity of the data”* (33)
Feedback and opportunities for personal development	VBHC resource	(9, 56, 57, 73)	“*Measuring outcomes and discussing them at an OCN [obstetric collaborative networks] level was considered to have the potential to stimulate learning”* (56)
	VBHC demand	(57, 73)	“*In the absence of such tools, participants perceived a lack of insight into their own care delivery, which was considered a real hindrance to critical refection on HV3C delivery and their ability to train residents in such behavior”* (57)
Pace of change and time availability	Environment demand	(14, 24, 33, 49, 50, 55, 60, 74)	“*[They] expressed the view that they were burdened by the pressure of time. Participants did not have time to anchor changes in work outside the pilot project team. It was more important to uphold the consultants' time plan than actually to allow enough time for related health personnel”* (24)
Data and IT	VBHC resource	(32, 50)	“*Experienced facilitators focus on the availability of individual, N = 1, PROMs scores, that could prepare both patients and professionals for discussion of patient values”* (50)
	Environment resource	(50, 61)	“*Advanced visualization of the bars and graphs of the PROMs scores (N = 1) [as facilitator]”* (50)
	VBHC demand	(50)	“*Lack of overview of all existing options for the specific patient groups, for example, regarding transmural care, rehabilitation, and primary care”* (50)
	Environment demand	(14, 24, 31–33, 35, 48–50, 62, 66, 69, 72, 76)	“*They also reported poor access to both quality data and cost data”* (48)
Authority and say	Environment demand	(14, 24, 33, 55, 76)	“*The lack of power within the implementation team to drive change”* (76)
Autonomy	VBHC demand	(73)	“*Physicians reported pressure to abide by CPW [clinical pathways] […] Participants expressed concern that CPW encourage providers to adhere to an algorithm or an outlined plan, which can stifle one's education by limiting critical-thinking skills and autonomy. CPW lead to ‘prescriptive medicine’ where care may be simplified too much”* (73)
	Environment demand	(24)	“*The high tempo during the first three months deprived the participants of their own autonomy”* (24)
Organizational support and legitimacy	VBHC resource	(24)	“*Even if it was impossible to make use of all the patient representatives' opinions and experiences, participants were proud of their cooperation with the representatives as this contributed to the legitimacy of their implementation work”* (24)
	Environment resource	(24, 33, 48, 50, 69)	“*Over time, participants came to understand the importance of the hospital director's unequivocal standpoint that VBHC was to be used as a management tool. This standpoint gave legitimacy to decisions within the teams”* (33)
	Environment demand	(24, 32, 33, 35, 55, 72, 76)	“*Participants felt they had been thrown into the deep end when it came to implementation work”* (33)
Pride	VBHC resource	(24)	“*[…] participants were proud of their cooperation with the representatives as this contributed to the legitimacy of their implementation work”* (24)

##### Role

VBHC itself and how organizations shaped VBHC impacted professionals' roles and interests both positively and negatively. VBHC can be considered a job resource as healthcare professionals mentioned that VBHC aligned with their interest, ethics, and nature of their work and reconnected them with their true role (24, 31, 35, 54, 55, 66, 72). Within VBHC, teams and workflows were reconfigured to allow everyone to utilize their competences to the full extent. However, when the reconfiguration was inadequate, professionals were concerned to become IT-specialists and were hindered to use their competences optimally (66). Consequently, professionals experienced job demands when their work environment did not support them to practice their role (33, 38, 55). VBHC itself also introduced role strain (14, 24, 32, 34, 51, 55, 60, 69, 74). For example, professionals found it hard to balance patient care and implementation work (33), questioned their role in discussing costs with patients (69), and experienced role unclarity due to new responsibilities in VBHC that were not yet formalized (14, 32). Residents in particular experienced specific strains related to priority-setting between VBHC and learning goals and felt uncertain about their contribution to VBHC (51, 55, 60, 74).

##### Workload and Time Investment

VBHC was suggested to take more time and effort than providing lower-value care and hence was considered a job demand (31–33, 51, 62, 66, 71, 73). Among others, providing preoperative services and continuous work on pathways were considered time consuming. Related to organizational facilities and resources in the work environment, inadequate data-systems were suggested to increase work burden by demanding more manual work (33). However, when workflow and team compositions were adequately shaped, professionals experienced reduced administrative workload (60, 66, 72). This suggests that VBHC can also turn into a job resource.

##### Competence

Although residents reported adequate VBHC knowledge (75) and nurses mentioned VBHC as one of their best competences (77, 79), the majority of studies revealed knowledge, skill, and experience deficits (31, 33, 38, 49, 50, 55, 57, 59, 60, 62, 75). These deficits related to, among others, tailoring care, managing case complexity, care integration and coordination, IT and data, quality improvement, interpretation and use of PROMs scores, exploring treatment options, benchmarking, knowledge about healthcare costs, and overall maintenance of knowledge.

##### Task Complexity

Two studies reported on increased task complexity in VBHC. One study mentioned that nurses experienced complexity with new tasks in VBHC as a result of task expansion (60). The second study suggested information overload due to working with care pathways (73).

##### Work Method

Professionals appreciated VBHC's contribution to easier, more effective and better structured ways of working (24, 31, 32, 49, 50). VBHC was mentioned to make patient follow-up easier, to bring more focus, specific tasks, and better insight in care processes. Moreover, VBHC was considered a tool for well-founded decisions and documentation (31, 32). However, professionals mentioned to lack an approach to quality improvement and felt hindered by pathways and guidelines that were inexplicit and difficult to access and interpret (14, 49, 73). Organizational structure and division of financial responsibilities were environmental factors experienced to obstruct care processes (24).

##### Meaning in Work

Participants experienced successes from their value-based efforts and increased sense of purpose and mission (24, 31, 32, 35, 56, 60, 61, 66, 73). Examples of successes were better care transitions, achievement of the Triple aim, reduction of low-value care, elimination of care variation, and overall improved care in favor of the patient. Visible effects were mentioned to be motivating, and when invisible this had negative impact on engagement (24, 31). Remarkably, one study reported that only half of the participants saw success from their efforts to promote quality care at lower cost (69). Furthermore, one study described that implementation work was seen as an “obligation” and considered meaningless in light of persisting root-cause problems in the organization (24). This experience was characterized as a job demand that stemmed from characteristics of the environment.

##### Patient Contact

Both beneficial and adverse outcomes of VBHC on patient contact were reported. On the one hand, VBHC was experienced to improve patient contact. In particular, PROMs prepared patients and professionals for discussing patient values (50), care pathways facilitated conversations with patients and families regarding plans of care (73), and patients perceived their professionals to be better informed as result from strengthened team-based care (61). On the other hand, professionals seemingly faced more challenges in value-based patient contact (49–51, 55, 57, 69, 73–75). Professionals reported difficulties, reluctance and discomfort when discussing VBHC with patients, specifically costs (48, 55, 57, 69), and the choice of non-treatment (50). Professionals also mentioned to face demanding patients and patients with wrong expectations, which hindered or even prevented them to provide VBHC (49, 51, 55, 75). Last, concern was expressed about pathways limiting patient discussions by creating “tunnel vision” (73).

##### Teamwork and Communication

VBHC created organizational imperative for professionals to cooperate and was considered to facilitate cooperation by providing a shared language. This resulted in the perception of more and better teamwork (24, 32, 44, 53, 61, 66, 73). However, collaboration between silos was mentioned to cause confusion (24). Prompted by the environment, participants felt it was difficult to maintain staff engagement, faced adverse behavior of colleagues, and reported on being tangled up in discussions about (im)possibilities regarding data collection (33, 47, 49, 51, 73).

##### Comfort and Confidence

While pathways enhanced confidence by affirming clinical decision-making (73), professionals also experienced lack of comfort and uncertainty in VBHC (48, 51, 55, 60, 69, 73, 75). Among others, professionals felt lack of comfort with the complexity of new tasks (60) and comfort with cost conversations varied (48, 51). Diagnostic uncertainty and concerns about inadequate patient follow-up were identified as reasons why professionals overuse resources (75). Professionals also felt insecure when they had to capture data manually due to IT limitations (33), being an environment-specific factor.

##### Feedback and Opportunities for Personal Development

VBHC education and training, as environmental factors, have not been included in this study. However, it is of interest to note that professionals reported on learning potential being stimulated by outcome information (9, 56), feedback tools (57), and pathways (73). However, professionals also recognized that pathways possibly limit learnings (73). Feedback tools were considered useful and when absent professionals experienced this as hindering (57).

##### Pace of Change and Time Availability

Participants felt pressured by time, especially due to the absence of dedicated time for VBHC activities and rapid pace of implementation (14, 24, 33, 49, 50, 55, 60, 74). Due to this pressure, participants felt deprived of their autonomy (24) and reported losing focus (55). They regretted not working up to their best (33) and fell back into care of lower value (74).

##### Data and IT

Professionals valued that VBHC provided access to PROMs scores of individual patients and patient codes (32, 50). Professionals appreciated work environments that provided advanced PROMs score visualizations and adequate access to the electronic health record (50, 61). Hindrance was experienced as a result of not having access to aggregated PROMs data and lacking overview of treatments options (50). Furthermore, various deficiencies related to data, IT, data collection routines, and infrastructure hindered professionals in pursuing VBHC (14, 24, 31–33, 35, 48–50, 62, 66, 69, 72, 76). These demanding situations were triggered by inadequate organizational structures and resources in the professional's work environment.

##### Authority and Say

Some professionals felt obstructed to participate in VBHC and drive VBHC as a team leader (14, 24, 33, 55, 76). This was caused by a lack of authority and say within their work environment. This lack was considered problematic as it hindered decision-making.

##### Autonomy

As a characteristic of VBHC, professionals experienced reduced autonomy due to the felt pressure to abide by pathways (73). As an environmental demand, professionals described being deprived of their autonomy due to rapid implementation of VBHC (24). Additionally, two studies reported on autonomy of professionals being purposefully adjusted in VBHC. One study increased professionals' autonomy to advance VBHC. In this study professionals were authorized to select their own performance metrics (23). In another study, autonomy of junior residents was reduced as they were seen as potential providers of lower value care and hence in need of guidance and limits (57).

##### Organizational Support and Legitimacy

Professionals experienced legitimacy in value-based work as a result of involving patient representatives (24), which was consequently considered a resource stemming from VBHC. There was variation to what extent professionals felt supported in their work environment. On the positive side, professionals described, among others, support from managers, leadership, and champions as role model (24, 33, 48, 50, 69). On the negative side, professionals described, among others, disinterest of managers, skepticism in IT departments and lack of, and unclear, policy (24, 32, 33, 35, 55, 72, 76). VBHC consultants and guidelines were mentioned to potentially be helpful but also risked to cause drawbacks when utilized inappropriately (24, 55).

##### Pride

A single study reported that the involvement of patient representatives made professionals experience pride (24).

#### Employee Well-Being and Job Strain

Positive and negative outcomes of VBHC for professionals were reported. These, as discussed below, related to employee well-being and job strain. Table 2 provides an overview.

**Table 2:** Overview and illustrative quotes on employee well-being and job strain in VBHC.

**Employee well-being**	**Studies**	**Exemplifying quote**
Engagement	(24, 35, 44, 60, 61)	“*The focus on value for the patient, emphasized by the hospital management team, contributed to their feelings of ‘enthusiasm for the concept and strong engagement in implementation work”* (24)
Engagement being moderated by demands	(24)	“*These hindrances contributed to decreasing engagement in carrying the process forward. […] Engagement for VBHC also decreased when participants did not see any actual activity or result of their implementation work”* (24)
Being energized	(24, 66)	“*I think even greater sense of meaning that we're all working towards the greater good of patient health and well-being, and I think that genuinely energized people”* (66)
Having joy in practice	(66)	“*All but one of the practices indicated that their transformation efforts led to increased joy of practice”* (66)
Joy in practice being moderated by demands	(66)	“*The one outlier practice indicated increased sense of purpose and mission and did not indicate decrease in joy or well-being, but did acknowledge that increased work necessary for practice transformation moderated increased joy of practice”* (66)
Increased Job Satisfaction	(24, 32, 44, 60, 66)	“*All participants in the structured interviews noted improved job satisfaction after the transition period, given the new sense of employee engagement and accountability”* (44)
**Job strain**	**Studies**	**Exemplifying quote**
Exhaustion and energy drain	(24, 32, 33)	“*This was experienced as a long and energy-draining process”* (32)
Negative emotions	(24, 33, 47, 55, 73)	“*Participants expressed both their colleagues and their nonadherence to CPW [clinical pathways] can result in a range of emotions from fear to frustration”* (73)
Losing focus and getting stuck	(24, 33)	“*In all, these residents sometimes let time pressure, demanding patients, concerns over supervisors potentially overruling them, their wish to develop or maintain a patient–resident relationship, and fears of claims make them lose their focus on HV3C delivery”* (55)
Concerns	(24, 31, 32, 48, 50, 51, 53, 55, 56, 69, 73–76)	“*Nearly 50% reported that the clinicians' fear of legal repercussions affects their frequency of ordering unneeded tests or procedures, and 30% reported that individual clinicians are blamed for complications”* (69)
Burnout	(38)	“*Those who felt burned out at the completion of training (β=-0.52, 95% CI −1.00– 0.04, p=0.03) were more likely to score lower on the [Residency High Value Care] scale”* (38)

##### Employee Well-Being

Related to employee well-being in VBHC, positive outcomes included professionals who were engaged (24, 35, 44, 60, 61), felt energized (24, 66), experienced joy in practice (66), and experienced improved job satisfaction (24, 32, 44, 60, 66). These outcomes were suggested to positively impact subsequent VBHC behaviors (24, 35, 61).

Job resources associated with aforementioned positive outcomes were “role fit,” “work method,” and “meaning in work.” Professionals valued being able to focus on what matters to patients, working on specific tasks, seeing effects of their efforts, having outcomes to demonstrate, and meeting the Triple aim (24, 66). Positive outcomes also resulted from working in line with standard care plans (32), team-based care (66), redesigned workflows (60), multidisciplinary rounds with an experienced physician as coach (61), and practice transformation (44, 66).

Of interest, two studies reported that engagement and joy in practice were moderated or reduced by job demands. Job demands that decreased engagement were “role strain,” i.e., professionals who felt divided between different obligations, and “lack of meaning”, i.e., professional who did not see visible results from their VBHC efforts (24). The job demand that decreased joy in practice was increased “workload” (66).

##### Job Strain

Concerning job strain in VBHC, professionals experienced four negative outcomes, namely: exhaustion and energy drain (24, 32, 33), negative emotions (24, 33, 47, 55, 73), losing focus and getting stuck (24, 33), and several concerns (24, 31, 32, 48, 50, 51, 53, 55, 56, 69, 73–76). Negative emotions comprised frustration, fear, and feelings of guilt. Concerns related to care quality, VBHC continuity, pathways use, legal repercussions in combination with use of outcomes, hierarchy, and sustainability of the care system. A single study investigated burn-out as an input variable, showing that residents who felt burned out after their education scored lower on the “high-value care culture” scale (38).

Exhaustion and energy drain was associated with the job demand inadequate “data and IT.” Negative emotions were triggered by the job demands lack of “available time,” “teamwork” challenges, “role strain,” and inadequate “data and IT” including professionals' inabilities to change the IT system. Negative emotions also resulted from staffing constraints, hindering organizational structures and were associated with possible adverse consequences of pathways. Participants lost their focus and mentioned to risk not being able to uphold VBHC due the job demands “role strain,” insufficient “organizational support,” inadequate “pace of change and time availability,” challenging “patient contact,” meso-level obstacles and because of various concerns professionals had concerning VBHC.

## Discussion

The founders of VBHC state that professionals play a crucial role in VBHC and hence argue that employee well-being should be part of organizations' imperatives in addition to improving health value (1). However, to date, knowledge about what VBHC means for healthcare professionals is scattered. This review synthesizes insights from 45 included studies about how VBHC relates to the healthcare professional, and vice versa.

This review shows that the term VBHC is used for a variety of value-enhancing activities. Consequently, behaviors of professionals in VBHC may be specific to the type of activity performed. Thematic analysis reveals 10 specific behaviors that healthcare professionals pursue in VBHC, next to acting upon their professional standards. These interconnected and mutually reinforcing behaviors, as visualized in Figure 4, are to (1) focus on what matters to patients & adopt other VBHC mindsets, (2) measure outcomes, (3) learn and improve care, (4) organize care around the full cycle of disease, (5) participate in population health and prevention, (6) discuss value in the clinical encounter, (7) involve patient representatives, (8) take accountability for patients and resources, (9) practice bottom-up engagement, and above all (10) work in teams and collaborate.

### Job Demands-Resources in VBHC

This review confirms that VBHC “brings change to the current landscape by introducing new or different roles for people, different workflows or processes, and new tools or existing ones that have been used in other settings or all the above” (65). These changes impact the job demands and resources professionals experience in VBHC and, in turn, their well-being and job strain. More specifically, this review reveals that healthcare professionals in VBHC may experience 16 job resources and/or job demands, four constructs related to their well-being, and five constructs related to job strain. Figure 3 visualizes these outcomes in a modified Job Demands-Resources (JD-R) model.

Among others, the identified job resources suggest that VBHC connects professionals with their role and interest, making them appreciate VBHC as an approach to caring. Professionals report on increased meaning in their work and improved patient contact, teamwork, and communication. However, implementation of VBHC also takes energy from professionals. Although some studies report on reduced administrative workload in VBHC, other studies suggest that VBHC increases workload. This difference, as well as how other work factors are evaluated, may be partly explained by variety in professionals' work environments such as the level of organizational support, as elaborated below. Other job demands professionals may experience are role strain, teething problems with the transformation to VBHC and overall challenges evoked by change. Furthermore, within their organization, professionals seem to experience paucity of adequate IT resources, authority to implement VBHC and time to become acquainted with VBHC. Professionals also report on difficulties in discussing costs with patients. The latter is striking as we do not find literature that advises professionals to discuss costs with patients as part of VBHC besides themselves taking accountability for adequate use of resources. Hence, this disparity may suggest that the job demand that relates to discussing costs with patients is redundant.

This review reveals that increased job resources resulting from the adoption of VBHC may increase professionals' engagement, energy, joy in practice, and job satisfaction, which corresponds to findings from research on clinician engagement during organizational change (80). Respectively, job demands professionals experience in VBHC can make them feel exhausted and evoke negative emotions, loss of focus and concerns. This review reveals that job demands may moderate employee engagement and joy in practice, as has also been suggested in JD-R literature (81). The positive effect of job resources on job strain that this literature describes is not explicitly mentioned in the included studies of this review. Remarkably, the included studies only qualitatively investigate employee well-being and exhaustion while quantitative measurement instruments exist, for example as part of the JD-R questionnaire (82).

Altogether, the aforementioned job demands, job resources and outcomes related to employee well-being and job strain show similarities with earlier research on job demands and resources in healthcare setting (41, 80) albeit sometimes in slightly different wording. This may imply that VBHC involves various established psychosocial factors at work and not so much radically introduces new factors that seek our attention. However, the results from this review may be too rosy as VBHC projects to date possibly focused on low-hanging fruits. Moreover, the identified factors may apply to specific VBHC components and be partly environment specific. This implies that the results from this review are not expected to apply to all professionals and hence should be interpreted with care.

### Organizational Support as Enabler

The strength of this review is that it distinguishes between job resources and job demands that stem from (1) VBHC in terms of content and (2) the environment in which VBHC takes place. For example, professionals who experience that VBHC takes more effort is considered a demand that stems from VBHC. Professionals who feel pressure from the pace of implementation is considered a demand that stems from the work environment, as it depends on how organizations shape and facilitate VBHC. This distinction is in line with the concept of psychosocial factors at work, which explicitly distinguishes between job content, work environment, and organizational conditions as factors that impact employee well-being (13).

Strikingly, this review finds that several job demands stem from organizations' inadequate management of VBHC, i.e., speeded VBHC implementation, suboptimal workforce composition connected to care pathways and insufficient organizational resources and capacity. This observation underlines the need for organizations to better support their employees by providing the necessary resources and designing appropriate organizational structures and interventions to mitigate or avoid job demands and enhance job resources. Subsequently, this may sustainably improve professionals' contributions to VBHC via improved employee well-being. This is especially relevant in the light of research relating employee experience and well-being to organizational performance measures (83, 84) such as workforce engagement in healthcare development (85). In other words, just personal engagement of professionals is insufficient as is illustrated by the following quote: “[They] recognize that HV3C [high-value, cost-conscious care] practices depend in part on the patient population, available resources, and organizational structure […] Although they initially aimed to provide HV3C, under external pressure their pro-HV3C aspirations waned” (55).

The view that VBHC is a shared responsibility and requires multi-level support is supported by the adapted JOINT model (42). This model defines five layers, being the (1) individual layer, (2) interpersonal layer, (3) job level layer, (4) organizational layer, and (5) national layer. Each of these layers has been suggested to impact nurse absenteeism and turnover (42). Not only can multi-layered support help us reduce negative psychosocial work factors in VBHC and hence prevent disease and dysfunction in the workforce, but also can this layered support contribute positive psychosocial work factors in VBHC and hence support professionals to flourish. On the organizational level, support may be best shown to advantage as part of a “top-guided bottom-up” approach. In a top-guided bottom-up approach efforts of professionals, primarily teams, are orchestrated centrally (86). Within this approach organizations provide their employees supportive infrastructure, tools and resources including protected time, relevant data, staff training, and administrative and analytic support.

### Limitations

This study has five biases. First, the identified outcomes of VBHC on professionals' experiences and their well-being may not be generalizable to all professionals working in a VBHC context for three reasons. Namely, scholars may use different criteria for judging whether their intervention is part of VBHC, studies report on different combinations of VBHC activities and – as this review concludes – experiences may be partly work environment specific. A second bias is that studies reporting on high-value care and high-value, cost-conscious care are generalized while there may be subtle differences between these care models. Hence, we may expect professionals to pursue slightly different behaviors in each of these care models, which, in turn, may evoke slightly different experiences and outcomes. Third, this review does not distinguish between the type of healthcare professional and her educational status. Clinicians, nurses, and residents, who form the main populations in the included studies, may fulfill different roles in VBHC and hence can be expected to have different experiences and encounter different personal outcomes. Consequently, based on this review, it is not possible to target focused interventions to specific populations. Fourth, the temporality of the findings is uncertain as some experiences and outcomes may be connected to implementation efforts more than being a lasting characteristic of VBHC. However, judging whether VBHC has become part of the normal work is complicated as this perception is suggested to vary from professional to professional (24). Last, assessing whether a job demand or resource is a characteristic of VBHC or a characteristic of the environment is a delicate task and requires certain interpretability as all care activities take place in an environment. This implies that different takes on the resulting overview of job demands and resources are possible.

### Practical Implications

Prompted by the insight that healthcare professionals may experience paucity of competence to optimally pursue value in care, we identify the need for more guidance for professionals. Providing adequate guidance is especially relevant as professionals play a prominent role in VBHC (1), which aligns with our findings. Moreover, value-enhancing behaviors of professionals, such as shared decision making, increasingly become legal requirements (87, 88). The 10 behaviors this review describes (see Figure 4) may serve as a base for this guidance. While some of these behaviors correspond to Porter's value agenda (89), this review also proposes new behaviors. In line with an earlier proposed extension to Porter's value agenda (7), this review suggests to incorporate behaviors to “learn and improve care” and to “discuss value in the clinical encounter” as additional elements. Furthermore, this review focusses attention to the need for professionals to “adopt appropriate mindsets for VBHC,” in particular by truly focusing on what matters to patients. Other behaviors this review contributes are to “work in teams and collaborate,” “involve patient representatives,” “take accountability for patients and resources,” “practice bottom-up engagement,” and “participate in population health and prevention.”

Besides guidance for professionals, this review also supports organizations to better care for their employees and strive for a sustainable VBHC model. This review shows how organizations can use a psychosocial model such as JD-R to manage and improve employee well-being, as has been previously suggested to Human Resource Management as well (HRM) (83, 90). Caring for employees is besides being morally integer and beneficial for organizational performance also a legal obligation in Europe (91). In addition to mitigating and avoiding adverse effects of VBHC on the professional, organizations may seek to exploit VBHC to contribute to positive psychosocial factors at work. For example, organizations may amplify job resources such as “meaning in work” by enhancing the visibility of VBHC outcomes.

As previously mentioned, organizations can consider a top-guided bottom-up approach (86) to optimally support their employees in VBHC. Within this approach, attention should be given to the pre-implementation and delivery phase of VBHC to prevent professionals from having avoidable adverse experiences. The International Labour Organisation (13) studied frequent omissions and mistakes when implementing changes at the workplace. From this research we derive that technical and psychological preparation is needed prior to implementation. For VBHC this implies that, among others, PROM technologies and care pathways should be adequately established and professionals need to be sufficiently informed and trained. Second, during VBHC delivery, professionals should be offered support depending on their personal needs. Next to the use of PROMs and PREMs, we see opportunity to periodically evaluate psychosocial factors at work and use these results for improvements. Third, organizational should give explicit attention to implementing VBHC at a satisfying pace in the eyes of professionals since professionals reported to feel pressured. Furthermore, organizations need to ensure that professionals have necessary authority to implement and deliver VBHC as professional mentioned lack of authority as impediment to VBHC. Last, by preventing staff shortages, providing professionals dedicated time for VBHC and optimizing team composition, organizations can mitigate or avoid increases in professionals' workload and even exploit VBHC to reduce administrative workload and optimize job resources such as meaning in work, comfort and collaboration.

### Future Work

Contributions of this study to literature are two-fold. First this work contributes to JD-R literature by considering that job demands and resources may both result from the nature of the job and the way actors in the environment facilitate and shape the job. Future work using the JD-R model may want to explicitly research the antecedents of job demands and resources as this allows for focusing interventions at the source. Antecedents identified in prior research on psychosocial factors at work may provide inspiration (13). Second, this work contributes to VBHC literature by shifting attention toward the professional. This review reveals several behaviors professionals pursue to achieve value in care, job demands and resources professionals experience in VBHC and, in turn, outcomes related to employee well-being and job strain.

Further research to estimate the effects of VBHC on healthcare professionals is warranted. First, application of existing theories and frameworks is recommended as only one of the studies included in this review did so. Second, this review provides an overview of factors that impact the professional and her delivery of VBHC both positively and negatively. Future work may investigate sufficient and necessary conditions to make VBHC work such as strong leadership, a culture of continuous improvement and strengthened team-based care. Third, future work may focus on personal resources in VBHC as these seem understudied. Personal resources, such as optimism and self-efficacy, may affect a person's functioning and are hence integrated in the JD-R model (92). Another opportunity for future work focuses on pre-existing care practices that gained a new life in VBHC, such as efforts to improve care and working with PROMs. This review builds on the assumption that these care practices are experienced differently now they are applied as mechanisms to optimize value in care as opposed to satisfying different purposes or being an end-goal in themselves. However, future research is necessary to validate this assumption. Finally, due to the multifaceted nature of VBHC, scholars may attempt to study how, and to what degree, each component of VBHC, as well as possible interactions between components, impacts job experience and employee well-being. Impact evaluations of VBHC implementation programs across different hospitals would allow to generate such insights among healthcare professionals. The ongoing transformation from traditional healthcare delivery to VBHC provides momentum for evaluation of the effectiveness of VBHC in relation to job experience and employee well-being by comparing traditional care practices to value-based care practices.

## Data Availability Statement

The original contributions presented in the study are included in the article/Supplementary Material, further inquiries can be directed to the corresponding author.

## Author Contributions

VE led the development of the search strategy, screened all papers (first screener), led the analyses, and wrote the first draft of the manuscript. IB was involved in conceptualization of the study, development of the search strategy, screening of the papers (second screener), analyses, and reviewed the manuscript. KA was involved in conceptualization of the study, development of the search strategy, analyses, and reviewed the manuscript. MB-S led the conceptualization of the study, was involved in development of the search strategy, screening of a subset of papers (third screener), analyses, and reviewed the manuscript. All authors approved the final version of the manuscript.

## Funding

This work was funded by Erasmus School of Health Policy & Management, Erasmus University Rotterdam.

## Conflict of Interest

The authors declare that the research was conducted in the absence of any commercial or financial relationships that could be construed as a potential conflict of interest.

## Publisher's Note

All claims expressed in this article are solely those of the authors and do not necessarily represent those of their affiliated organizations, or those of the publisher, the editors and the reviewers. Any product that may be evaluated in this article, or claim that may be made by its manufacturer, is not guaranteed or endorsed by the publisher.
