# Supplementary material for: Value-Based Healthcare From the Perspective of the Healthcare Professional: A Systematic Literature Review
Source: Front Public Health. 2022 Jan 13;9:800702. doi: 10.3389/fpubh.2021.800702 (PMC8792751; doi:10.3389/fpubh.2021.800702)
Supplement: Supplementary file 1 [file Data_Sheet_1.docx]

Search string

# Additional information

- The two groups of the International Standard Classification of Occupations ISCO-08 (International Labour Office, 2012) that were included are 1) 'health professionals' (medical doctors and nursing professionals) and 2) the group ‘other care professionals’ (e.g. such as dentists, pharmacists and dieticians). These groups cover most care-related occupations.
- Human values with a double meaning, e.g. power, have been excluded as inclusion would increase the number of retrieved studies substantially.

# Results per database

| Database | Number of studies | Number of studies after removal of duplicates |
| --- | --- | --- |
| Embase.com | 1020 | 1000 |
| Medline ALL Ovid | 974 | 120 |
| PsycINFO ALL Ovid | 303 | 203 |
| Web of Science (SCI-EXPANDED & SSCI) | 524 | 201 |
| CINAHL EBSCOhost | 806 | 286 |
| Business Source Premier EBSCOhost | 150 | 88 |
| EconLit ProQuest | 5 | 5 |
| Total | 3782 | 1903 |

# Search strings

## Embase.com

('value based care'/de OR'value based medicine'/de OR (vbhc OR vb-hc OR ((value-based OR valuebased) NOT ((value-based OR valuebased) NEXT/2 (insuran* OR purchas* OR pric* OR reimburse* OR contract* OR payment* OR partnership*))) OR ((high-value OR value-driven) NEAR/3 (care OR healthcare))):ab,ti) AND ('health personnel attitude'/expOR 'professional competence'/de OR 'job satisfaction'/de OR 'job satisfaction assessment'/de OR (('health care personnel'/exp OR workplace/exp OR 'work environment'/exp) AND ('personal experience'/de OR wellbeing/de OR satisfaction/de OR 'clinical competence'/de OR morality/de OR perception/de OR courage/de OR leadership/de OR motivation/de OR cooperation/de OR 'behavior change'/exp OR 'coping behavior'/de)) OR (((personnel* OR Physician* OR Specialist* OR Medical-Specialist* OR Medical-assistant* OR Nurse* OR Doctor* OR Anesthesiologist* OR Anaesthesiologist* OR Cardiologist* OR Dermatologist* OR Endocrinologist* OR Geriatrician* OR Gynecologist* OR Gynaecologist* OR Hematologist* OR Haematologist* OR Neurologist* OR Obstetrician* OR Oncologist* OR Otolaryngologist* OR Optometrist* OR Pediatrician* OR Paediatrician* OR Pathologist* OR Pulmonologist* OR Radiologist* OR Rheumatologist* OR Surgeon* OR Traumatologist* OR Urologist* OR General-Practioner* OR GP* OR Family-doctor* OR Therapist* OR Dentist* OR Physiotherapist* OR Physical-therapist* OR Dietician* OR Pharmacist* OR Psychologist* OR Psychiatrist* OR professional* OR provider* OR job OR workplaceOR work-place OR clinician* OR staff* OR member* OR workforce* OR work-force* OR team OR teams) NEAR/6 (attitude* OR involvement* OR Fulfillment* OR Engagement* OR Involvement* OR Commitment* OR Motivat* OR Intention* OR Behav* OR Belief* OR View* OR Perspective* OR Perception* OR Opinion* OR Feeling* OR Experience* OR Emotion* OR Satisf* OR Self-efficac* OR Qualities* OR Mindset* OR Mind-set* OR Virtue* OR Stress* OR Burn-out*OR Burnout* OR Teamwork* OR Team-work* OR Wellbeing* OR Values* OR Performance* OR Collaboration* OR Climate* OR Universalism* OR Equalit* OR Benevolence* OR Altruism* OR Conformit* OR Tradition* OR Moralit* OR Securit* OR Power* OR Authorit* OR Achievement* OR Capabilit* OR Hedonism* OR Pleasure* OR Self-direction* OR Critical-thinking* OR Stimulation* OR Humilit* OR Broadminded* OR Wisdom* OR Justice* OR Tolerance* OR Understand* OR Appreciation* OR Acceptance* OR Respect OR Advocacy* OR Equity* OR Dignity* OR Socialism* OR Solidarity* OR Humanism* OR Helping* OR Honest* OR Forgiving* OR Loyal* OR Responsible* OR Friendship* OR Love* OR Belonging* OR Meaning* OR Caring* OR Empathy* OR Compassion* OR Polite* OR Obedien* OR Self-disciplin* OR Honoring* OR Loyal* OR Responsib* OR Adheren* OR nonAdheren* OR complian* OR noncomplian* OR Fitting-in* OR Self-aware* OR Ethical-behav* OR Humble* OR Acceptan* OR Devout* OR Honour* OR Integrity* OR Honest* OR Morality* OR Duty* OR Duties* OR Temperan* OR Harmony* OR Stability* OR Social-order* OR Belonging* OR Relatedness* OR Confidentiality* OR Emotional-stability* OR Prudence* OR Vigilance* OR Self-protection* OR Trust* OR Well-paid* OR Financial-stability* OR Comfort* OR Convenien* OR Control* OR Dominan* OR Social-power* OR Recognition* OR Leader* OR lead OR Hierarch* OR Paternalism* OR Capab* OR Competen* OR Ambitious* OR Influential* OR Intelligence* OR Approval* OR Enjoy* OR Self-indulgen* OR Excite* OR Novelt* OR Challenge* OR Daring* OR Variation* OR Independen* OR Autonom* OR Creativit* OR Freedom* OR Curious* OR Goal-setting* OR Problem-solving* OR Imagination* OR Objectivity* OR Self-regulation* OR Status* OR Prestige* OR Humble* OR Self-effac* OR Safety OR FaceOR Moderate* OR Wealth OR Success* OR Image OR moral OR capacit* OR courage* OR self-report* OR participat* OR cooperat* OR workload*OR work-load* OR proactiv*OR pro-activ* OR Influence* OR Impact* OR Affect* OR Effect* OR Outcome* OR Implication* OR Consequence* OR Change* OR Contribute* OR Benefit* OR Advantage* OR Disadvantage* OR Drawback* OR Driver* OR Tension* OR Conflict* OR learning OR atmosphere* OR culture* OR cohesion* OR development* OR think OR cope OR coping)) OR Professionalism*):ab,ti) NOT [conference abstract]/lim

## Medline ALL Ovid

((vbhc OR vb-hc OR ((value-based OR valuebased) NOT ((value-based OR valuebased) ADJ2 (insuran* OR purchas* OR pric* OR reimburse* OR contract* OR payment* OR partnership*))) OR ((high-value OR value-driven) ADJ3 (care OR healthcare))).ab,ti.) AND (Attitude of Health Personnel / OR Professional Competence / OR Job Satisfaction/ OR ((expHealth Personnel / OR Workplace/) AND (Personal Satisfaction/ OR Clinical Competence/ OR Morals / OR Perception/ OR Courage/ OR Leadership/ OR Motivation/ OR Adaptation, Psychological /)) OR (((personnel* OR Physician* OR Specialist* OR Medical-Specialist* OR Medical-assistant* OR Nurse* OR Doctor* OR Anesthesiologist* OR Anaesthesiologist* OR Cardiologist* OR Dermatologist* OR Endocrinologist* OR Geriatrician* OR Gynecologist* OR Gynaecologist* OR Hematologist* OR Haematologist* OR Neurologist* OR Obstetrician* OR Oncologist* OR Otolaryngologist* OR Optometrist* OR Pediatrician* OR Paediatrician* OR Pathologist* OR Pulmonologist* OR Radiologist* OR Rheumatologist* OR Surgeon* OR Traumatologist* OR Urologist* OR General-Practioner* OR GP* OR Family-doctor* OR Therapist* OR Dentist* OR Physiotherapist* OR Physical-therapist* OR Dietician* OR Pharmacist* OR Psychologist* OR Psychiatrist* OR professional* OR provider* OR job OR workplaceOR work-place OR clinician* OR staff* OR member* OR workforce* OR work-force* OR team OR teams) ADJ6 (attitude* OR involvement* OR Fulfillment* OR Engagement* OR Involvement* OR Commitment* OR Motivat* OR Intention* OR Behav* OR Belief* OR View* OR Perspective* OR Perception* OR Opinion* OR Feeling* OR Experience* OR Emotion* OR Satisf* OR Self-efficac* OR Qualities* OR Mindset* OR Mind-set* OR Virtue* OR Stress* OR Burn-out*OR Burnout* OR Teamwork* OR Team-work* OR Wellbeing* OR Values* OR Performance* OR Collaboration* OR Climate* OR Universalism* OR Equalit* OR Benevolence* OR Altruism* OR Conformit* OR Tradition* OR Moralit* OR Securit* OR Power* OR Authorit* OR Achievement* OR Capabilit* OR Hedonism* OR Pleasure* OR Self-direction* OR Critical-thinking* OR Stimulation* OR Humilit* OR Broadminded* OR Wisdom* OR Justice* OR Tolerance* OR Understand* OR Appreciation* OR Acceptance* OR Respect OR Advocacy* OR Equity* OR Dignity* OR Socialism* OR Solidarity* OR Humanism* OR Helping* OR Honest* OR Forgiving* OR Loyal* OR Responsible* OR Friendship* OR Love* OR Belonging* OR Meaning* OR Caring* OR Empathy* OR Compassion* OR Polite* OR Obedien* OR Self-disciplin* OR Honoring* OR Loyal* OR Responsib* OR Adheren* OR nonAdheren* OR complian* OR noncomplian* OR Fitting-in* OR Self-aware* OR Ethical-behav* OR Humble* OR Acceptan* OR Devout* OR Honour* OR Integrity* OR Honest* OR Morality* OR Duty* OR Duties* OR Temperan* OR Harmony* OR Stability* OR Social-order* OR Belonging* OR Relatedness* OR Confidentiality* OR Emotional-stability* OR Prudence* OR Vigilance* OR Self-protection* OR Trust* OR Well-paid* OR Financial-stability* OR Comfort* OR Convenien* OR Control* OR Dominan* OR Social-power* OR Recognition* OR Leader* OR lead OR Hierarch* OR Paternalism* OR Capab* OR Competen* OR Ambitious* OR Influential* OR Intelligence* OR Approval* OR Enjoy* OR Self-indulgen* OR Excite* OR Novelt* OR Challenge* OR Daring* OR Variation* OR Independen* OR Autonom* OR Creativit* OR Freedom* OR Curious* OR Goal-setting* OR Problem-solving* OR Imagination* OR Objectivity* OR Self-regulation* OR Status* OR Prestige* OR Humble* OR Self-effac* OR Safety OR FaceOR Moderate* OR Wealth OR Success* OR Image OR moral OR capacit* OR courage* OR self-report* OR participat* OR cooperat* OR workload*OR work-load* OR proactiv*OR pro-activ* OR Influence* OR Impact* OR Affect* OR Effect* OR Outcome* OR Implication* OR Consequence* OR Change* OR Contribute* OR Benefit* OR Advantage* OR Disadvantage* OR Drawback* OR Driver* OR Tension* OR Conflict* OR learning OR atmosphere* OR culture* OR cohesion* OR development* OR think OR cope OR coping)) OR Professionalism*).ab,ti.)

## PsycINFO ALL Ovid

((vbhc OR vb-hc OR ((value-based OR valuebased) NOT ((value-based OR valuebased) ADJ2 (insuran* OR purchas* OR pric* OR reimburse* OR contract* OR payment* OR partnership*))) OR ((high-value OR value-driven) ADJ3 (care OR healthcare))).ab,ti.) AND (Health Personnel Attitudes / OR Professional Competence / OR Job Satisfaction/ OR ((expHealth Personnel/) AND (Satisfaction/ OR Professional Competence / OR Morality/ OR Courage/ OR Leadership/ OR Motivation/ OR Adaptation / OR Emotional Adjustment/)) OR (((personnel* OR Physician* OR Specialist* OR Medical-Specialist* OR Medical-assistant* OR Nurse* OR Doctor* OR Anesthesiologist* OR Anaesthesiologist* OR Cardiologist* OR Dermatologist* OR Endocrinologist* OR Geriatrician* OR Gynecologist* OR Gynaecologist* OR Hematologist* OR Haematologist* OR Neurologist* OR Obstetrician* OR Oncologist* OR Otolaryngologist* OR Optometrist* OR Pediatrician* OR Paediatrician* OR Pathologist* OR Pulmonologist* OR Radiologist* OR Rheumatologist* OR Surgeon* OR Traumatologist* OR Urologist* OR General-Practioner* OR GP* OR Family-doctor* OR Therapist* OR Dentist* OR Physiotherapist* OR Physical-therapist* OR Dietician* OR Pharmacist* OR Psychologist* OR Psychiatrist* OR professional* OR provider* OR job OR workplaceOR work-place OR clinician* OR staff* OR member* OR workforce* OR work-force* OR team OR teams) ADJ6 (attitude* OR involvement* OR Fulfillment* OR Engagement* OR Involvement* OR Commitment* OR Motivat* OR Intention* OR Behav* OR Belief* OR View* OR Perspective* OR Perception* OR Opinion* OR Feeling* OR Experience* OR Emotion* OR Satisf* OR Self-efficac* OR Qualities* OR Mindset* OR Mind-set* OR Virtue* OR Stress* OR Burn-out*OR Burnout* OR Teamwork* OR Team-work* OR Wellbeing* OR Values* OR Performance* OR Collaboration* OR Climate* OR Universalism* OR Equalit* OR Benevolence* OR Altruism* OR Conformit* OR Tradition* OR Moralit* OR Securit* OR Power* OR Authorit* OR Achievement* OR Capabilit* OR Hedonism* OR Pleasure* OR Self-direction* OR Critical-thinking* OR Stimulation* OR Humilit* OR Broadminded* OR Wisdom* OR Justice* OR Tolerance* OR Understand* OR Appreciation* OR Acceptance* OR Respect OR Advocacy* OR Equity* OR Dignity* OR Socialism* OR Solidarity* OR Humanism* OR Helping* OR Honest* OR Forgiving* OR Loyal* OR Responsible* OR Friendship* OR Love* OR Belonging* OR Meaning* OR Caring* OR Empathy* OR Compassion* OR Polite* OR Obedien* OR Self-disciplin* OR Honoring* OR Loyal* OR Responsib* OR Adheren* OR nonAdheren* OR complian* OR noncomplian* OR Fitting-in* OR Self-aware* OR Ethical-behav* OR Humble* OR Acceptan* OR Devout* OR Honour* OR Integrity* OR Honest* OR Morality* OR Duty* OR Duties* OR Temperan* OR Harmony* OR Stability* OR Social-order* OR Belonging* OR Relatedness* OR Confidentiality* OR Emotional-stability* OR Prudence* OR Vigilance* OR Self-protection* OR Trust* OR Well-paid* OR Financial-stability* OR Comfort* OR Convenien* OR Control* OR Dominan* OR Social-power* OR Recognition* OR Leader* OR lead OR Hierarch* OR Paternalism* OR Capab* OR Competen* OR Ambitious* OR Influential* OR Intelligence* OR Approval* OR Enjoy* OR Self-indulgen* OR Excite* OR Novelt* OR Challenge* OR Daring* OR Variation* OR Independen* OR Autonom* OR Creativit* OR Freedom* OR Curious* OR Goal-setting* OR Problem-solving* OR Imagination* OR Objectivity* OR Self-regulation* OR Status* OR Prestige* OR Humble* OR Self-effac* OR Safety OR FaceOR Moderate* OR Wealth OR Success* OR Image OR moral OR capacit* OR courage* OR self-report* OR participat* OR cooperat* OR workload*OR work-load* OR proactiv*OR pro-activ* OR Influence* OR Impact* OR Affect* OR Effect* OR Outcome* OR Implication* OR Consequence* OR Change* OR Contribute* OR Benefit* OR Advantage* OR Disadvantage* OR Drawback* OR Driver* OR Tension* OR Conflict* OR learning OR atmosphere* OR culture* OR cohesion* OR development* OR think OR cope OR coping)) OR Professionalism*).ab,ti.)

## Web of Science (SCI-EXPANDED & SSCI)

TS=(((vbhc OR vb-hc OR ((value-based OR valuebased) NOT ((value-based OR valuebased) NEAR/2 (insuran* OR purchas* OR pric* OR reimburse* OR contract* OR payment* OR partnership*))) OR ((high-value OR value-driven) NEAR/2 (care OR healthcare)))) AND ((((healthcare-personnel* OR health-care-personnel* OR Physician* OR Specialist* OR Medical-Specialist* OR Medical-assistant* OR Nurse* OR Doctor* OR Anesthesiologist* OR Anaesthesiologist* OR Cardiologist* OR Dermatologist* OR Endocrinologist* OR Geriatrician* OR Gynecologist* OR Gynaecologist* OR Hematologist* OR Haematologist* OR Neurologist* OR Obstetrician* OR Oncologist* OR Otolaryngologist* OR Optometrist* OR Pediatrician* OR Paediatrician* OR Pathologist* OR Pulmonologist* OR Radiologist* OR Rheumatologist* OR Surgeon* OR Traumatologist* OR Urologist* OR General-Practioner* OR GP OR Family-doctor* OR Therapist* OR Dentist* OR Physiotherapist* OR Physical-therapist* OR Dietician* OR Pharmacist* OR Psychologist* OR Psychiatrist* OR healthcare-professional* OR health-care-professional* OR healthcare-provider* OR health-care-provider*) NEAR/5 (attitude* OR involvement* OR Fulfillment* OR Engagement* OR Involvement* OR Commitment* OR Motivat* OR Intention* OR Behav* OR Belief* OR View* OR Perspective* OR Perception* OR Opinion* OR Feeling* OR Experience* OR Emotion* OR Satisf* OR Self-efficac* OR Qualities* OR Mindset* OR Mind-set* OR Virtue* OR Stress* OR Burn-out*OR Burnout* OR Teamwork* OR Team-work* OR Wellbeing* OR Values* OR Performance* OR Collaboration* OR Climate* OR Universalism* OR Equalit* OR Benevolence* OR Altruism* OR Conformit* OR Tradition* OR Moralit* OR Securit* OR Power* OR Authorit* OR Achievement* OR Capabilit* OR Hedonism* OR Pleasure* OR Self-direction* OR Critical-thinking* OR Stimulation* OR Humilit* OR Broadminded* OR Wisdom* OR Justice* OR Tolerance* OR Understand* OR Appreciation* OR Acceptance* OR Respect OR Advocacy* OR Equity* OR Dignity* OR Socialism* OR Solidarity* OR Humanism* OR Helping* OR Honest* OR Forgiving* OR Loyal* OR Responsible* OR Friendship* OR Love* OR Belonging* OR Meaning* OR Caring* OR Empathy* OR Compassion* OR Polite* OR Obedien* OR Self-disciplin* OR Honoring* OR Loyal* OR Responsib* OR Adheren* OR nonAdheren* OR complian* OR noncomplian* OR Fitting-in* OR Self-aware* OR Ethical-behav* OR Humble* OR Acceptan* OR Devout* OR Honour* OR Integrity* OR Honest* OR Morality* OR Duty* OR Duties* OR Temperan* OR Harmony* OR Stability* OR Social-order* OR Belonging* OR Relatedness* OR Confidentiality* OR Emotional-stability* OR Prudence* OR Vigilance* OR Self-protection* OR Trust* OR Well-paid* OR Financial-stability* OR Comfort* OR Convenien* OR Control* OR Dominan* OR Social-power* OR Recognition* OR Leader* OR lead OR Hierarch* OR Paternalism* OR Capab* OR Competen* OR Ambitious* OR Influential* OR Intelligence* OR Approval* OR Enjoy* OR Self-indulgen* OR Excite* OR Novelt* OR Challenge* OR Daring* OR Variation* OR Independen* OR Autonom* OR Creativit* OR Freedom* OR Curious* OR Goal-setting* OR Problem-solving* OR Imagination* OR Objectivity* OR Self-regulation* OR Status* OR Prestige* OR Humble* OR Self-effac* OR Safety OR FaceOR Moderate* OR Wealth OR Success* OR Image OR moral OR capacit* OR courage* OR self-report* OR participat* OR cooperat* OR workload*OR work-load* OR proactiv*OR pro-activ* OR Influence* OR Impact* OR Affect* OR Effect* OR Outcome* OR Implication* OR Consequence* OR Change* OR Contribute* OR Benefit* OR Advantage* OR Disadvantage* OR Drawback* OR Driver* OR Tension* OR Conflict* OR learning OR atmosphere* OR culture* OR cohesion* OR development* OR think OR cope OR copingOR Professionalism*))))) AND DT=(article)

## CINAHL EBSCOhost

((TI(vbhc OR vb-hcOR ((high-value OR value-driven) N2 (care OR healthcare))) OR AB(vbhc OR vb-hcOR ((high-value OR value-driven) N2 (care OR healthcare))) OR ((TI(value-based OR valuebased) OR AB(value-based OR valuebased)) NOT (TI((value-based OR valuebased) N2 (insuran* OR purchas* OR pric* OR reimburse* OR contract* OR payment* OR partnership*)) OR AB((value-based OR valuebased) N2 (insuran* OR purchas* OR pric* OR reimburse* OR contract* OR payment* OR partnership*)))))) AND (MH Attitude of Health Personnel + OR MH Professional Competence OR MH Job Satisfaction OR ((MH Health Personnel + OR MH Work Environment ) AND (MH Personal Satisfaction OR MH Clinical Competence OR MH Morals OR MH Perception OR MH Courage OR MH Leadership OR MH Motivation OR MH Adaptation, Psychological)) OR TI(((personnel* OR Physician* OR Specialist* OR Medical-Specialist* OR Medical-assistant* OR Nurse* OR Doctor* OR Anesthesiologist* OR Anaesthesiologist* OR Cardiologist* OR Dermatologist* OR Endocrinologist* OR Geriatrician* OR Gynecologist* OR Gynaecologist* OR Hematologist* OR Haematologist* OR Neurologist* OR Obstetrician* OR Oncologist* OR Otolaryngologist* OR Optometrist* OR Pediatrician* OR Paediatrician* OR Pathologist* OR Pulmonologist* OR Radiologist* OR Rheumatologist* OR Surgeon* OR Traumatologist* OR Urologist* OR General-Practioner* OR GP* OR Family-doctor* OR Therapist* OR Dentist* OR Physiotherapist* OR Physical-therapist* OR Dietician* OR Pharmacist* OR Psychologist* OR Psychiatrist* OR professional* OR provider* OR job OR workplaceOR work-place OR clinician* OR staff* OR member* OR workforce* OR work-force* OR team OR teams) N5 (attitude* OR involvement* OR Fulfillment* OR Engagement* OR Involvement* OR Commitment* OR Motivat* OR Intention* OR Behav* OR Belief* OR View* OR Perspective* OR Perception* OR Opinion* OR Feeling* OR Experience* OR Emotion* OR Satisf* OR Self-efficac* OR Qualities* OR Mindset* OR Mind-set* OR Virtue* OR Stress* OR Burn-out*OR Burnout* OR Teamwork* OR Team-work* OR Wellbeing* OR Values* OR Performance* OR Collaboration* OR Climate* OR Universalism* OR Equalit* OR Benevolence* OR Altruism* OR Conformit* OR Tradition* OR Moralit* OR Securit* OR Power* OR Authorit* OR Achievement* OR Capabilit* OR Hedonism* OR Pleasure* OR Self-direction* OR Critical-thinking* OR Stimulation* OR Humilit* OR Broadminded* OR Wisdom* OR Justice* OR Tolerance* OR Understand* OR Appreciation* OR Acceptance* OR Respect OR Advocacy* OR Equity* OR Dignity* OR Socialism* OR Solidarity* OR Humanism* OR Helping* OR Honest* OR Forgiving* OR Loyal* OR Responsible* OR Friendship* OR Love* OR Belonging* OR Meaning* OR Caring* OR Empathy* OR Compassion* OR Polite* OR Obedien* OR Self-disciplin* OR Honoring* OR Loyal* OR Responsib* OR Adheren* OR nonAdheren* OR complian* OR noncomplian* OR Fitting-in* OR Self-aware* OR Ethical-behav* OR Humble* OR Acceptan* OR Devout* OR Honour* OR Integrity* OR Honest* OR Morality* OR Duty* OR Duties* OR Temperan* OR Harmony* OR Stability* OR Social-order* OR Belonging* OR Relatedness* OR Confidentiality* OR Emotional-stability* OR Prudence* OR Vigilance* OR Self-protection* OR Trust* OR Well-paid* OR Financial-stability* OR Comfort* OR Convenien* OR Control* OR Dominan* OR Social-power* OR Recognition* OR Leader* OR lead OR Hierarch* OR Paternalism* OR Capab* OR Competen* OR Ambitious* OR Influential* OR Intelligence* OR Approval* OR Enjoy* OR Self-indulgen* OR Excite* OR Novelt* OR Challenge* OR Daring* OR Variation* OR Independen* OR Autonom* OR Creativit* OR Freedom* OR Curious* OR Goal-setting* OR Problem-solving* OR Imagination* OR Objectivity* OR Self-regulation* OR Status* OR Prestige* OR Humble* OR Self-effac* OR Safety OR FaceOR Moderate* OR Wealth OR Success* OR Image OR moral OR capacit* OR courage* OR self-report* OR participat* OR cooperat* OR workload*OR work-load* OR proactiv*OR pro-activ* OR Influence* OR Impact* OR Affect* OR Effect* OR Outcome* OR Implication* OR Consequence* OR Change* OR Contribute* OR Benefit* OR Advantage* OR Disadvantage* OR Drawback* OR Driver* OR Tension* OR Conflict* OR learning OR atmosphere* OR culture* OR cohesion* OR development* OR think OR cope OR coping)) OR Professionalism*)OR AB(((personnel* OR Physician* OR Specialist* OR Medical-Specialist* OR Medical-assistant* OR Nurse* OR Doctor* OR Anesthesiologist* OR Anaesthesiologist* OR Cardiologist* OR Dermatologist* OR Endocrinologist* OR Geriatrician* OR Gynecologist* OR Gynaecologist* OR Hematologist* OR Haematologist* OR Neurologist* OR Obstetrician* OR Oncologist* OR Otolaryngologist* OR Optometrist* OR Pediatrician* OR Paediatrician* OR Pathologist* OR Pulmonologist* OR Radiologist* OR Rheumatologist* OR Surgeon* OR Traumatologist* OR Urologist* OR General-Practioner* OR GP* OR Family-doctor* OR Therapist* OR Dentist* OR Physiotherapist* OR Physical-therapist* OR Dietician* OR Pharmacist* OR Psychologist* OR Psychiatrist* OR professional* OR provider* OR job OR workplaceOR work-place OR clinician* OR staff* OR member* OR workforce* OR work-force* OR team OR teams) N5 (attitude* OR involvement* OR Fulfillment* OR Engagement* OR Involvement* OR Commitment* OR Motivat* OR Intention* OR Behav* OR Belief* OR View* OR Perspective* OR Perception* OR Opinion* OR Feeling* OR Experience* OR Emotion* OR Satisf* OR Self-efficac* OR Qualities* OR Mindset* OR Mind-set* OR Virtue* OR Stress* OR Burn-out*OR Burnout* OR Teamwork* OR Team-work* OR Wellbeing* OR Values* OR Performance* OR Collaboration* OR Climate* OR Universalism* OR Equalit* OR Benevolence* OR Altruism* OR Conformit* OR Tradition* OR Moralit* OR Securit* OR Power* OR Authorit* OR Achievement* OR Capabilit* OR Hedonism* OR Pleasure* OR Self-direction* OR Critical-thinking* OR Stimulation* OR Humilit* OR Broadminded* OR Wisdom* OR Justice* OR Tolerance* OR Understand* OR Appreciation* OR Acceptance* OR Respect OR Advocacy* OR Equity* OR Dignity* OR Socialism* OR Solidarity* OR Humanism* OR Helping* OR Honest* OR Forgiving* OR Loyal* OR Responsible* OR Friendship* OR Love* OR Belonging* OR Meaning* OR Caring* OR Empathy* OR Compassion* OR Polite* OR Obedien* OR Self-disciplin* OR Honoring* OR Loyal* OR Responsib* OR Adheren* OR nonAdheren* OR complian* OR noncomplian* OR Fitting-in* OR Self-aware* OR Ethical-behav* OR Humble* OR Acceptan* OR Devout* OR Honour* OR Integrity* OR Honest* OR Morality* OR Duty* OR Duties* OR Temperan* OR Harmony* OR Stability* OR Social-order* OR Belonging* OR Relatedness* OR Confidentiality* OR Emotional-stability* OR Prudence* OR Vigilance* OR Self-protection* OR Trust* OR Well-paid* OR Financial-stability* OR Comfort* OR Convenien* OR Control* OR Dominan* OR Social-power* OR Recognition* OR Leader* OR lead OR Hierarch* OR Paternalism* OR Capab* OR Competen* OR Ambitious* OR Influential* OR Intelligence* OR Approval* OR Enjoy* OR Self-indulgen* OR Excite* OR Novelt* OR Challenge* OR Daring* OR Variation* OR Independen* OR Autonom* OR Creativit* OR Freedom* OR Curious* OR Goal-setting* OR Problem-solving* OR Imagination* OR Objectivity* OR Self-regulation* OR Status* OR Prestige* OR Humble* OR Self-effac* OR Safety OR FaceOR Moderate* OR Wealth OR Success* OR Image OR moral OR capacit* OR courage* OR self-report* OR participat* OR cooperat* OR workload*OR work-load* OR proactiv*OR pro-activ* OR Influence* OR Impact* OR Affect* OR Effect* OR Outcome* OR Implication* OR Consequence* OR Change* OR Contribute* OR Benefit* OR Advantage* OR Disadvantage* OR Drawback* OR Driver* OR Tension* OR Conflict* OR learning OR atmosphere* OR culture* OR cohesion* OR development* OR think OR cope OR coping)) OR Professionalism*))

## Business Source Premier EBSCOhost

((TI(vbhc OR vb-hcOR ((high-value OR value-driven) N2 (care OR healthcare))) OR AB(vbhc OR vb-hcOR ((high-value OR value-driven) N2 (care OR healthcare))) OR ((TI(value-based OR valuebased) OR AB(value-based OR valuebased)) NOT (TI((value-based OR valuebased) N2 (insuran* OR purchas* OR pric* OR reimburse* OR contract* OR payment* OR partnership*)) OR AB((value-based OR valuebased) N2 (insuran* OR purchas* OR pric* OR reimburse* OR contract* OR payment* OR partnership*)))))) AND (MH HOSPITAL personnel attitudes+ OR ((MH MEDICAL personnel+ OR MH Work Environment ) AND (MH EMPLOYEE morale OR MH Leadership OR MH EMPLOYEE motivation OR MH Job Satisfaction)) OR TI(((healthcare-personnel* OR health-care-personnel* OR Physician* OR Specialist* OR Medical-Specialist* OR Medical-assistant* OR Nurse* OR Doctor* OR Anesthesiologist* OR Anaesthesiologist* OR Cardiologist* OR Dermatologist* OR Endocrinologist* OR Geriatrician* OR Gynecologist* OR Gynaecologist* OR Hematologist* OR Haematologist* OR Neurologist* OR Obstetrician* OR Oncologist* OR Otolaryngologist* OR Optometrist* OR Pediatrician* OR Paediatrician* OR Pathologist* OR Pulmonologist* OR Radiologist* OR Rheumatologist* OR Surgeon* OR Traumatologist* OR Urologist* OR General-Practioner* OR GP* OR Family-doctor* OR Therapist* OR Dentist* OR Physiotherapist* OR Physical-therapist* OR Dietician* OR Pharmacist* OR Psychologist* OR Psychiatrist* OR healthcare-professional* OR health-care-professional* OR healthcare-provider* OR health-care-provider*) N5 (attitude* OR involvement* OR Fulfillment* OR Engagement* OR Involvement* OR Commitment* OR Motivat* OR Intention* OR Behav* OR Belief* OR View* OR Perspective* OR Perception* OR Opinion* OR Feeling* OR Experience* OR Emotion* OR Satisf* OR Self-efficac* OR Qualities* OR Mindset* OR Mind-set* OR Virtue* OR Stress* OR Burn-out*OR Burnout* OR Teamwork* OR Team-work* OR Wellbeing* OR Values* OR Performance* OR Collaboration* OR Climate* OR Universalism* OR Equalit* OR Benevolence* OR Altruism* OR Conformit* OR Tradition* OR Moralit* OR Securit* OR Power* OR Authorit* OR Achievement* OR Capabilit* OR Hedonism* OR Pleasure* OR Self-direction* OR Critical-thinking* OR Stimulation* OR Humilit* OR Broadminded* OR Wisdom* OR Justice* OR Tolerance* OR Understand* OR Appreciation* OR Acceptance* OR Respect OR Advocacy* OR Equity* OR Dignity* OR Socialism* OR Solidarity* OR Humanism* OR Helping* OR Honest* OR Forgiving* OR Loyal* OR Responsible* OR Friendship* OR Love* OR Belonging* OR Meaning* OR Caring* OR Empathy* OR Compassion* OR Polite* OR Obedien* OR Self-disciplin* OR Honoring* OR Loyal* OR Responsib* OR Adheren* OR nonAdheren* OR complian* OR noncomplian* OR Fitting-in* OR Self-aware* OR Ethical-behav* OR Humble* OR Acceptan* OR Devout* OR Honour* OR Integrity* OR Honest* OR Morality* OR Duty* OR Duties* OR Temperan* OR Harmony* OR Stability* OR Social-order* OR Belonging* OR Relatedness* OR Confidentiality* OR Emotional-stability* OR Prudence* OR Vigilance* OR Self-protection* OR Trust* OR Well-paid* OR Financial-stability* OR Comfort* OR Convenien* OR Control* OR Dominan* OR Social-power* OR Recognition* OR Leader* OR lead OR Hierarch* OR Paternalism* OR Capab* OR Competen* OR Ambitious* OR Influential* OR Intelligence* OR Approval* OR Enjoy* OR Self-indulgen* OR Excite* OR Novelt* OR Challenge* OR Daring* OR Variation* OR Independen* OR Autonom* OR Creativit* OR Freedom* OR Curious* OR Goal-setting* OR Problem-solving* OR Imagination* OR Objectivity* OR Self-regulation* OR Status* OR Prestige* OR Humble* OR Self-effac* OR Safety OR FaceOR Moderate* OR Wealth OR Success* OR Image OR moral OR capacit* OR courage* OR self-report* OR participat* OR cooperat* OR workload*OR work-load* OR proactiv*OR pro-activ* OR Influence* OR Impact* OR Affect* OR Effect* OR Outcome* OR Implication* OR Consequence* OR Change* OR Contribute* OR Benefit* OR Advantage* OR Disadvantage* OR Drawback* OR Driver* OR Tension* OR Conflict* OR learning OR atmosphere* OR culture* OR cohesion* OR development* OR think OR cope OR coping)) OR Professionalism*)OR AB(((healthcare-personnel* OR health-care-personnel* OR Physician* OR Specialist* OR Medical-Specialist* OR Medical-assistant* OR Nurse* OR Doctor* OR Anesthesiologist* OR Anaesthesiologist* OR Cardiologist* OR Dermatologist* OR Endocrinologist* OR Geriatrician* OR Gynecologist* OR Gynaecologist* OR Hematologist* OR Haematologist* OR Neurologist* OR Obstetrician* OR Oncologist* OR Otolaryngologist* OR Optometrist* OR Pediatrician* OR Paediatrician* OR Pathologist* OR Pulmonologist* OR Radiologist* OR Rheumatologist* OR Surgeon* OR Traumatologist* OR Urologist* OR General-Practioner* OR GP* OR Family-doctor* OR Therapist* OR Dentist* OR Physiotherapist* OR Physical-therapist* OR Dietician* OR Pharmacist* OR Psychologist* OR Psychiatrist* OR healthcare-professional* OR health-care-professional* OR healthcare-provider* OR health-care-provider*) N5 (attitude* OR involvement* OR Fulfillment* OR Engagement* OR Involvement* OR Commitment* OR Motivat* OR Intention* OR Behav* OR Belief* OR View* OR Perspective* OR Perception* OR Opinion* OR Feeling* OR Experience* OR Emotion* OR Satisf* OR Self-efficac* OR Qualities* OR Mindset* OR Mind-set* OR Virtue* OR Stress* OR Burn-out*OR Burnout* OR Teamwork* OR Team-work* OR Wellbeing* OR Values* OR Performance* OR Collaboration* OR Climate* OR Universalism* OR Equalit* OR Benevolence* OR Altruism* OR Conformit* OR Tradition* OR Moralit* OR Securit* OR Power* OR Authorit* OR Achievement* OR Capabilit* OR Hedonism* OR Pleasure* OR Self-direction* OR Critical-thinking* OR Stimulation* OR Humilit* OR Broadminded* OR Wisdom* OR Justice* OR Tolerance* OR Understand* OR Appreciation* OR Acceptance* OR Respect OR Advocacy* OR Equity* OR Dignity* OR Socialism* OR Solidarity* OR Humanism* OR Helping* OR Honest* OR Forgiving* OR Loyal* OR Responsible* OR Friendship* OR Love* OR Belonging* OR Meaning* OR Caring* OR Empathy* OR Compassion* OR Polite* OR Obedien* OR Self-disciplin* OR Honoring* OR Loyal* OR Responsib* OR Adheren* OR nonAdheren* OR complian* OR noncomplian* OR Fitting-in* OR Self-aware* OR Ethical-behav* OR Humble* OR Acceptan* OR Devout* OR Honour* OR Integrity* OR Honest* OR Morality* OR Duty* OR Duties* OR Temperan* OR Harmony* OR Stability* OR Social-order* OR Belonging* OR Relatedness* OR Confidentiality* OR Emotional-stability* OR Prudence* OR Vigilance* OR Self-protection* OR Trust* OR Well-paid* OR Financial-stability* OR Comfort* OR Convenien* OR Control* OR Dominan* OR Social-power* OR Recognition* OR Leader* OR lead OR Hierarch* OR Paternalism* OR Capab* OR Competen* OR Ambitious* OR Influential* OR Intelligence* OR Approval* OR Enjoy* OR Self-indulgen* OR Excite* OR Novelt* OR Challenge* OR Daring* OR Variation* OR Independen* OR Autonom* OR Creativit* OR Freedom* OR Curious* OR Goal-setting* OR Problem-solving* OR Imagination* OR Objectivity* OR Self-regulation* OR Status* OR Prestige* OR Humble* OR Self-effac* OR Safety OR FaceOR Moderate* OR Wealth OR Success* OR Image OR moral OR capacit* OR courage* OR self-report* OR participat* OR cooperat* OR workload*OR work-load* OR proactiv*OR pro-activ* OR Influence* OR Impact* OR Affect* OR Effect* OR Outcome* OR Implication* OR Consequence* OR Change* OR Contribute* OR Benefit* OR Advantage* OR Disadvantage* OR Drawback* OR Driver* OR Tension* OR Conflict* OR learning OR atmosphere* OR culture* OR cohesion* OR development* OR think OR cope OR coping)) OR Professionalism*))

## EconLit ProQuest

TI,AB((vbhc OR vb-hc OR ((value-based OR valuebased) NOT ((value-based OR valuebased) N/2 (insuran* OR purchas* OR pric* OR reimburse* OR contract* OR payment* OR partnership*))) OR ((high-value OR value-driven) N/2 (care OR healthcare)))) AND TI,AB((((healthcare-personnel* OR health-care-personnel* OR Physician* OR Nurse* OR Doctor* OR healthcare-professional* OR health-care-professional* OR healthcare-provider* OR health-care-provider*))))
