# Supplementary material for: Value-Based Healthcare From the Perspective of the Healthcare Professional: A Systematic Literature Review
Source: Front Public Health. 2022 Jan 13;9:800702. doi: 10.3389/fpubh.2021.800702 (PMC8792751; doi:10.3389/fpubh.2021.800702)
Supplement: Supplementary file 2 [file Data_Sheet_2.docx]

Quality appraisal using the Mixed Method Appraisal Tool (MMAT)

# How MMAT has been used to appraise quality

- All included studies have been subjected to the two generic screening items (see below).
- Qualitative and quantitative studies have been subjected to their unique category of screening items (see below).
- Mixed methods studies have been subjected to all three screening categories (qualitative, quantitative and mixed method) (see below).
- Response options were: Yes, No and Can’t tell.

# Response to score conversion

As suggested in the MMAT 2018 guideline (Hong et al, 2018), ‘Yes’ responses have been scored ‘1’ and ‘No’ and ‘Can’t tell’ responses have been scored ‘0’. Each category can receive a maximum score of 5. The score ‘5’ means that 100% of the quality criteria are met. Respectively, a score of 0 means that none of the quality criteria were met. For mixed method studies the category with the lowest scoring assessment was used, because studies cannot exceed the quality of its weakest component.

MMAT does not provide cut off values to characterize ‘low’, ‘medium’ and ‘high’ quality studies. Authors are free in this choice as long as the chosen cutoff values are transparent. In this review three categories (low, medium, and high quality) were used, representing studies with scores 0-2, 3 and 4-5 respectively.

# Screening items (Hong et al, 2018)

## Screening (all studies)

S1. Are there clear research questions?

S2. Do the collected data allow to address the research questions?

## Qualitative

1.1. Is the qualitative approach appropriate to answer the research question?

1.2. Are the qualitative data collection methods adequate to address the research question?

1.3. Are the findings adequately derived from the data?

1.4. Is the interpretation of results sufficiently substantiated by data?

1.5. Is there coherence between qualitative data sources, collection, analysis and interpretation?

## Quantitative descriptive

4.1. Is the sampling strategy relevant to address the research question?

4.2. Is the sample representative of the target population?

4.3. Are the measurements appropriate?

4.4. Is the risk of nonresponse bias low?

4.5. Is the statistical analysis appropriate to answer the research question?

## Mixed methods

5.1. Is there an adequate rationale for using a mixed methods design to address the research question?

5.2. Are the different components of the study effectively integrated to answer the research question?

5.3. Are the outputs of the integration of qualitative and quantitative components adequately interpreted?

5.4. Are divergences and inconsistencies between quantitative and qualitative results adequately addressed?

5.5. Do the different components of the study adhere to the quality criteria of each tradition of the methods involved?

# Results

All included studies rated ‘Yes’ to the two screening questions, indicating that MMAT can be used as a tool for further assessment. These screening questions were left out in the table below.

| Study | Criteria from the Mixed Method Appraisal Tool (MMAT 2018) | | | | | | | | | | | | | | | Score | Quality |
| --- | --- | --- | --- | --- | --- | --- | --- | --- | --- | --- | --- | --- | --- | --- | --- | --- | --- |
|  | 1.1 | 1.2 | 1.3 | 1.4 | 1.5 | 4.1 | 4.2 | 4.3 | 4.4 | 4.5 | 5.1 | 5.2 | 5.3 | 5.4 | 5.5 |  |  |
| Qualitative designs | | | | | | | | | | | | | | | | | |
| Nilsson, K., Bååthe, F., Andersson, A. E., Wikström, E., & Sandoff, M. (2017). Experiences from implementing value-based healthcare at a Swedish University Hospital - an longitudinal interview study. BMC Health Services Research, 17(169), 1–12. https://doi.org/10.1186/s12913-017-2104-8 | 1 | 1 | 1 | 1 | 1 |  |  |  |  |  |  |  |  |  |  | 5 | High |
| Gross, D. J., Kennedy, M., Kothari, T., Scamurra, D. O., Wilkerson, M. L., Crawford, J. M., & Cohen, M. B. (2019). The Role of the Pathologist in Population Health. Archives of Pathology & Laboratory Medicine, 143, 610–620. https://doi.org/10.5858/arpa.2018-0223-CP | 1 | 0 | 0 | 0 | 0 |  |  |  |  |  |  |  |  |  |  | 1 | Low |
| Stammen, L., Slootweg, I., Stalmeijer, R., Janssen, L., Stassen, L., Scheele, F., & Driessen, E. (2019). The Struggle Is Real: How Residents Learn to Provide High-Value, Cost-Conscious Care. Teaching and Learning in Medicine, 31(4), 402–411. https://doi.org/10.1080/10401334.2019.1583566 | 1 | 1 | 1 | 1 | 1 |  |  |  |  |  |  |  |  |  |  | 5 | High |
| Nilsson, K., Bååthe, F., Erichsen Andersson, A., & Sandoff, M. (2018). The need to succeed – learning experiences resulting from the implementation of value-based healthcare. Leadership in Health Services, 31(1), 2–16. https://doi.org/10.1108/LHS-08-2016-0039 | 1 | 1 | 1 | 1 | 1 |  |  |  |  |  |  |  |  |  |  | 5 | High |
| Busari, J. O., & Duits, A. J. (2015). The strategic role of competency based medical education in health care reform: a case report from a small scale, resource limited, Caribbean setting. BMC Research Notes, 8(13), 1–8. https://doi.org/10.1186/s13104-014-0963-1 | 1 | 1 | 0 | 0 | 0 |  |  |  |  |  |  |  |  |  |  | 2 | Low |
| Dainty, K. N., Golden, B. R., Hannam, R., Webster, F., Browne, G., Mittmann, N., Stern, A., & Zwarenstein, M. (2018). A realist evaluation of value-based care delivery in home care: The influence of actors, autonomy and accountability. Social Science and Medicine, 206, 100–109. https://doi.org/10.1016/j.socscimed.2018.04.006 | 1 | 1 | 1 | 1 | 1 |  |  |  |  |  |  |  |  |  |  | 5 | High |
| Stammen, L. A., Driessen, E. W., Notermans, C. C. V. I., Scheele, F., Stassen, L. P. S., & Stalmeijer, R. E. (2020). How Do Attending Physicians Prepare Residents to Deliver High-Value, Cost-Conscious Care? Academic Medicine, 95, 764–770. https://doi.org/10.1097/acm.0000000000003051 | 1 | 1 | 1 | 1 | 1 |  |  |  |  |  |  |  |  |  |  | 5 | High |
| Tartaglia, K. M., Kman, N., & Ledford, C. (2015). Medical Student Perceptions of Cost-Conscious Care in an Internal Medicine Clerkship: A Thematic Analysis. Journal of General Internal Medicine. https://doi.org/10.1007/s11606-015-3324-4 | 0 | 0 | 1 | 1 | 1 |  |  |  |  |  |  |  |  |  |  | 3 | Medium |
| Nilsson, K., Bååthe, F., Erichsen Andersson, A., & Sandoff, M. (2017). Value-based healthcare as a trigger for improvement initiatives. Leadership in Health Services, 30(4), 364–377. https://doi.org/10.1108/LHS-09-2016-0045 | 1 | 1 | 1 | 1 | 1 |  |  |  |  |  |  |  |  |  |  | 5 | High |
| Blayney, D. W., Simon, M. K., Podtschaske, B., Ramsey, S., Shyu, M., Lindquist, C., & Milstein, A. (2018). Critical lessons from high-value oncology practices. JAMA Oncology, 4(2), 164–171. https://doi.org/10.1001/jamaoncol.2017.3803 | 1 | 1 | 1 | 1 | 1 |  |  |  |  |  |  |  |  |  |  | 5 | High |
| Meyer, M. (2017). Qualifications and Competencies for Population Health Management Positions: A Content Analysis of Job Postings. Population Health Management Heath Manage, 0(0), 475–485. https://doi.org/10.1089/pop.2016.0197 | 1 | 1 | 1 | 1 | 1 |  |  |  |  |  |  |  |  |  |  | 5 | High |
| Varley, A. L., Kripalani, S., Spain, T., Mixon, A. S., Acord, E., Rothman, R., & Limper, H. M. (2020). Understanding Factors Influencing Quality Improvement Capacity Among Ambulatory Care Practices Across the MidSouth Region: An Exploratory Qualitative Study. Quality Management in Health Care, 29(3), 136–141. https://doi.org/10.1097/qmh.0000000000000255 | 1 | 1 | 1 | 1 | 1 |  |  |  |  |  |  |  |  |  |  | 5 | High |
| Robinson, C., Lee, J. W., Davis, K. N., & O’Connor, M. (2019). Findings From FMAHealth’s Bright Spots in Practice Transformation Project. Family Medicine, 51(2), 137–142. https://doi.org/10.22454/FamMed.2019.163860 | 1 | 1 | 1 | 1 | 0 |  |  |  |  |  |  |  |  |  |  | 4 | High |
| Colldén, C., Gremyr, I., Hellström, A., & Sporraeus, D. (2017). A value-based taxonomy of improvement approaches in healthcare. Journal of Health, Organisation and Management, 31(4), 445–458. https://doi.org/10.1108/JHOM-08-2016-0162 | 1 | 1 | 1 | 1 | 1 |  |  |  |  |  |  |  |  |  |  | 5 | High |
| Steinmann, G., Van De Bovenkamp, H., De Bont, A., & Delnoij, D. (2020). Redefining value: a discourse analysis on value-based health care. BMC Health Services Research, 20(862), 1–13. https://doi.org/10.1186/s12913-020-05614-7 | 1 | 1 | 1 | 1 | 1 |  |  |  |  |  |  |  |  |  |  | 5 | High |
| O’Hara, K., Tanverdi, M., Reich, J., Scudamore, D. D., Tyler, A., & Bakel, L. A. (2020). Qualitative Study to Understand Pediatric Hospitalists and Emergency Medicine Physicians’ Perspectives of Clinical Pathways. Pediatric Quality & Safety, 5(2), 1–6. https://doi.org/10.1097/pq9.0000000000000270 | 1 | 1 | 1 | 1 | 1 |  |  |  |  |  |  |  |  |  |  | 5 | High |
| Demiris, G., Hodgson, N. A., Sefcik, J. S., Travers, J. L., McPhillips, M. V., & Naylor, M. D. (2020). High-value care for older adults with complex care needs: Leveraging nurses as innovators. Nursing Outlook, 68, 26–32. https://doi.org/10.1016/j.outlook.2019.06.019 | 1 | 0 | 0 | 0 | 0 |  |  |  |  |  |  |  |  |  |  | 1 | Low |
| Damman, O. C., Jani, A., de Jong, B. A., Becker, A., Metz, M. J., de Bruijne, M. C., Timmermans, D. R., Cornel, M. C., Ubbink, D. T., van der Steen, M., Gray, M., & van El, C. (2020). The use of PROMs and shared decision-making in medical encounters with patients: An opportunity to deliver value-based health care to patients. Journal of Evaluation in Clinical Practice, 26, 524–540. https://doi.org/10.1111/jep.13321 | 1 | 0 | 0 | 1 | 1 |  |  |  |  |  |  |  |  |  |  | 3 | Medium |
| Cornell, T. (2020). Leadership skills essential in the value-based care era. Leadership in Health Services, 33(3), 307–323. https://doi.org/10.1108/LHS-12-2019-0079 | 1 | 1 | 1 | 1 | 1 |  |  |  |  |  |  |  |  |  |  | 5 | High |
| Erichsen Andersson, A., Bååthe, F., Wikström, E., & Nilsson, K. (2015). Understanding value-based healthcare – an interview study with project team members at a Swedish university hospital. Journal of Hospital Administration, 4(4), 64–72. https://doi.org/10.5430/jha.v4n4p64 | 1 | 1 | 1 | 1 | 1 |  |  |  |  |  |  |  |  |  |  | 5 | High |
| Nilsson, K., & Sandoff, M. (2017). Leading implementation of the management innovation value-based healthcare at a Swedish University Hospital. Journal of Hospital Administration, 6(1), 51–59. https://doi.org/10.5430/jha.v6n1p51 | 1 | 1 | 1 | 1 | 1 |  |  |  |  |  |  |  |  |  |  | 5 | High |
| McAlearney, A. S., Walker, D. M., & Hefner, J. L. (2018). Moving Organizational Culture from Volume to Value: A Qualitative Analysis of Private Sector Accountable Care Organization Development. Health Services Research, 53(6), 4767–4788. https://doi.org/10.1111/1475-6773.13012 | 1 | 1 | 1 | 1 | 1 |  |  |  |  |  |  |  |  |  |  | 5 | High |
| Colldén, C., & Hellström, A. (2018). Value-based healthcare translated: A complementary view of implementation. BMC Health Services Research, 18(681), 1–12. https://doi.org/10.1186/s12913-018-3488-9 | 1 | 1 | 1 | 1 | 1 |  |  |  |  |  |  |  |  |  |  | 5 | High |
| Quantitative designs | | | | | | | | | | | | | | | | | |
| Makdisse, M., Ramos, P., Malheiro, D., Felix, M., Cypriano, A., Soares, J., Carneiro, A., Cendoroglo Neto, M., & Klajner, S. (2020). What Do Doctors Think About Value-Based Healthcare? A Survey of Practicing Physicians in a Private Healthcare Provider in Brazil. Value in Health Regional Issues, 23(C), 25–29. https://doi.org/10.1016/j.vhri.2019.10.003 |  |  |  |  |  | 1 | 1 | 0 | 0 | 1 |  |  |  |  |  | 3 | Medium |
| Beck, J. B., McDaniel, C. E., Bradford, M. C., Brock, D., Sy, C. D., Chen, T., Foti, J., & White, A. A. (2018). Prospective observational study on high-value care topics discussed on multidisciplinary rounds. Hospital Pediatrics, 8(3), 119–126. https://doi.org/10.1542/hpeds.2017-0183 |  |  |  |  |  | 0 | 0 | 1 | 0 | 1 |  |  |  |  |  | 2 | Low |
| Larsen, K. N., Kristensen, S. R., & Søgaard, R. (2018). Autonomy to health care professionals as a vehicle for value-based health care? Results of a quasi-experiment in hospital governance. Social Science and Medicine, 196, 37–46. https://doi.org/10.1016/j.socscimed.2017.11.009 |  |  |  |  |  | 1 | 0 | 0 | 1 | 1 |  |  |  |  |  | 3 | Medium |
| Gupta, R., Steers, N., Moriates, C., & Ong, M. (2018). Association between hospitalist productivity payments and high-value care culture. Journal of Hospital Medicine, 13, 16–21. https://doi.org/10.12788/jhm.3084 |  |  |  |  |  | 1 | 0 | 1 | 0 | 1 |  |  |  |  |  | 3 | Medium |
| Leep Hunderfund, A. N., Starr, S. R., Dyrbye, L. N., Baxley, E. G., Gonzalo, J. D., Miller, B. M., George, P., Morgan, H. K., Allen, B. L., Hoffman, A., Fancher, T. L., Mandrekar, J., & Reed, D. A. (2019). Imprinting on Clinical Rotations: Multisite Survey of High- and Low-Value Medical Student Behaviors and Relationship with Healthcare Intensity. Journal of General Internal Medicine, 34(7), 1131–1138. https://doi.org/10.1007/s11606-019-04828-8 |  |  |  |  |  | 1 | 0 | 1 | 1 | 1 |  |  |  |  |  | 4 | High |
| Pereira, V., Gabriel, M. H., & Unruh, L. (2019). Multiyear Performance Trends Analysis of Primary Care Practices Demonstrating Patient-Centered Medical Home Transformation: An Observation of Quality Improvement Indicators among Outpatient Clinics. American Journal of Medical Quality, 34(2), 109–118. https://doi.org/10.1177/1062860618792301 |  |  |  |  |  | 1 | 0 | 1 | 1 | 1 |  |  |  |  |  | 4 | High |
| Hanak, M. A., McDevitt, C., & Dunham, D. P. (2017). Perceptions of Ambulatory Workflow Changes in an Academic Primary Care Setting. The Health Care Manager, 36(3), 261–266. https://doi.org/10.1097/hcm.0000000000000174 |  |  |  |  |  | 1 | 0 | 0 | 0 | 1 |  |  |  |  |  | 2 | Low |
| Gupta, R., Steers, N., Moriates, C., Wali, S., Braddock, C. H., & Ong, M. (2019). High-Value Care Culture Among the Future Physician Workforce in Internal Medicine. Academic Medicine, 94(9), 1347–1354. https://doi.org/10.1097/acm.0000000000002619 |  |  |  |  |  | 1 | 0 | 1 | 0 | 1 |  |  |  |  |  | 3 | Medium |
| Grosso, M. J., Courtney, P. M., Kerr, J. M., Della Valle, C. J., & Huddleston, J. I. (2020). Surgeons’ Preoperative Work Burden Has Increased Before Total Joint Arthroplasty: A Survey of AAHKS Members. The Journal of Arthroplasty, 1–5. https://doi.org/10.1016/j.arth.2020.01.079 |  |  |  |  |  | 1 | 1 | 0 | 0 | 1 |  |  |  |  |  | 3 | Medium |
| Mordang, S. B. R., Leep Hunderfund, A. N., Smeenk, F. W. J. M., Stassen, L. P. S., & Könings, K. D. (2020). High-Value, Cost-Conscious Care Attitudes in the Graduate Medical Education Learning Environment: Various Stakeholder Attitudes That Residents Misjudge. Journal of General Internal Medicine. https://doi.org/10.1007/s11606-020-06261-8 |  |  |  |  |  | 0 | 1 | 1 | 0 | 1 |  |  |  |  |  | 3 | Medium |
| Brandt Vegas, D., Levinson, W., Norman, G., Monteiro, S., & You, J. J. (2015). Readiness of hospital-based internists to embrace and discuss high-value care with patients and family members: a single-centre cross-sectional survey study. CMAJ Open, 3(4), E382–E386. https://doi.org/10.9778/cmajo.20150024 |  |  |  |  |  | 1 | 0 | 1 | 0 | 1 |  |  |  |  |  | 3 | Medium |
| Nilsson, J., Mischo-Kelling, M., Thiekoetter, A., Deufert, D., Mendes, A. C., Fernandes, A., Kirchhoff, J. W., & Lepp, M. (2019). Nurse professional competence (NPC) assessed among newly graduated nurses in higher educational institutions in Europe. Nordic Journal of Nursing Research, 39(3), 159–167. https://doi.org/10.1177/2057158519845321 |  |  |  |  |  | 0 | 0 | 1 | 0 | 1 |  |  |  |  |  | 2 | Low |
| Gardulf, A., Florin, J., Carlsson, M., Leksell, J., Lepp, M., Lindholm, C., Nordström, G., Theander, K., Wilde-Larsson, B., & Nilsson, J. (2019). The Nurse Professional Competence (NPC) Scale: A tool that can be used in national and international assessments of nursing education programmes. Nordic Journal of Nursing Research, 0(0), 1–6. https://doi.org/10.1177/2057158518824530 |  |  |  |  |  | 1 | 0 | 1 | 0 | 1 |  |  |  |  |  | 3 | Medium |
| Ryskina, K. L., Smith, C. D., Weissman, A., Post, J., Dine, C. J., Bollmann, K. L., & Korenstein, D. (2015). U.S. Internal Medicine Residents’ Knowledge and Practice of High-Value Care: A National Survey. Academic Medicine, 90(10), 1373–1379. https://doi.org/10.1097/ACM.0000000000000791 |  |  |  |  |  | 1 | 1 | 0 | 0 | 1 |  |  |  |  |  | 3 | Medium |
| Mixed Method designs | | | | | | | | | | | | | | | | | |
| Artenstein, A. W., Higgins, T. L., Seiler, A., Meyer, D., Knee, A. B., Boynton, G., Picchioni, M., Geld, B., & Whitcomb, W. F. (2015). Promoting high value inpatient care via a coaching model of structured, interdisciplinary team rounds. British Journal of Hospital Medicine, 76(1), 41–45. https://doi.org/10.12968/hmed.2015.76.1.41 | 1 | 0 | 0 | 1 | 0 | 0 | 0 | 1 | 0 | 1 | 0 | 1 | 0 | 1 | 0 | 2 | Low |
| Ryskina, K. L., Holmboe, E. S., Shea, J. A., Kim, E., & Long, J. A. (2018). Physician Experiences with High Value Care in Internal Medicine Residency: Mixed Methods Study of 2003–2013 Residency Graduates. Teach Learn Medicine, 30(1), 57–66. https://doi.org/10.1080/10401334.2017.1335207 | 1 | 1 | 1 | 1 | 1 | 1 | 1 | 1 | 1 | 1 | 0 | 1 | 1 | 1 | 1 | 4 | High |
| Briggs, A. M., Houlding, E., Hinman, R. S., Desmond, L. A., Bennell, K. L., Darlow, B., Pizzari, T., Leech, M., MacKay, C., Larmer, P. J., Bendrups, A., Greig, A. M., Francis-Cracknell, A., Jordan, J. E., & Slater, H. (2019). Health professionals and students encounter multi-level barriers to implementing high-value osteoarthritis care: a multi-national study. Osteoarthritis Cartilage, 27, 788–804. https://doi.org/10.1016/j.joca.2018.12.024 | 1 | 1 | 1 | 1 | 1 | 1 | 1 | 1 | 0 | 1 | 1 | 1 | 1 | 1 | 1 | 4 | High |
| van Veghel, D., Daeter, E. J., Bax, M., Amoroso, G., Blaauw, Y., Camaro, C., Cummins, P., Halfwerk, F. R., Wijdh-Den Hamer, I. J., de Jong, J. S. S. G., Stooker, W., van der Wees, P. J., & van der Nat, P. B. (2019). Organization of outcome-based quality improvement in Dutch heart centres. European Heart Journal - Quality of Care and Clinical Outcomes, 0, 1–6. https://doi.org/10.1093/ehjqcco/qcz021 | 1 | 0 | 1 | 0 | 1 | 0 | 1 | 0 | 1 | 0 | 1 | 1 | 1 | 1 | 0 | 2 | Low |
| Sondheim, S. E., Patel, D. M., Chin, N., Barwis, K., Werner, J., Barclay, A., & Mattie, A. (2017). Governance Practices in an Era of Healthcare Transformation: Achieving a Successful Turnaround. Journal of Healthcare Management, 62(5), 316–326. https://doi.org/10.1097/jhm-d-15-00036 | 1 | 0 | 0 | 0 | 0 | 1 | 0 | 0 | 1 | 1 | 0 | 1 | 1 | 1 | 0 | 1 | Low |
| Laureij, L. T., Been, J. V., Lugtenberg, M., Ernst-Smelt, H. E., Franx, A., Hazelzet, J. A., de Groot, P. K., Frauenfelder, O., Henriquez, D., Lamain-de Ruiter, M., Neppelenbroek, E., Nij Bijvank, S. W. A., Schaap, T., Schagen, M., Veenhof, M., & Vermolen, J. H. (2020). Exploring the applicability of the pregnancy and childbirth outcome set: A mixed methods study. Patient Education and Counseling, 103, 642–651. https://doi.org/10.1016/j.pec.2019.09.022 | 1 | 1 | 1 | 1 | 1 | 0 | 1 | 1 | 0 | 1 | 1 | 1 | 1 | 1 | 1 | 3 | Medium |
| Wiencek, C. A., Kleinpell, R., Moss, M., & Sessler, C. N. (2019). Choosing Wisely in Critical Care: A National Survey of Critical Care Nurses. 28(6), 434–440. https://doi.org/10.4037/ajcc2019241 | 1 | 0 | 0 | 1 | 0 | 1 | 1 | 0 | 0 | 1 | 0 | 1 | 0 | 1 | 0 | 2 | Low |
| Boynes, S., Nelson, J., Diep, V., Kanan, C., Pedersen, D. N., Brown, C., Mathews, R., Tranby, E., Apostolon, D., Bayham, M., & Minter-Jordan, M. (2020). Understanding value in oral health: the oral health value-based care symposium. Journal of Public Health Dentistry, 80, S27–S34. https://doi.org/10.1111/jphd.12402 | 1 | 1 | 1 | 0 | 1 | 0 | 0 | 0 | 0 | 0 | 0 | 1 | 0 | 1 | 0 | 0 | Low |

# Results summary

| Study Design | Number of studies (total) | Number of studies per MMAT quality appraisal score category |
| --- | --- | --- |
| Qualitative | 23 | 18 High |
|  |  | 2 Medium |
|  |  | 3 Low |
| Quantitative | 14 | 2 High |
|  |  | 9 Medium |
|  |  | 3 Low |
| Mixed Method | 8 | 2 High |
|  |  | 1 Medium |
|  |  | 5 low |
