# Supplementary material for: Value-Based Healthcare From the Perspective of the Healthcare Professional: A Systematic Literature Review
Source: Front Public Health. 2022 Jan 13;9:800702. doi: 10.3389/fpubh.2021.800702 (PMC8792751; doi:10.3389/fpubh.2021.800702)
Supplement: Supplementary file 3 [file Data_Sheet_3.docx]

Summary table

| **Study** | **Authors** | **Year of publication** | **Country of studied population** | **Setting** | **Profession** | **Discipline/ specialty** | **Aim** | **Study design** | **VBHC taxonomy** | **Main VBHC interventions applied and/or studied** | **Involvement of the population in VBHC intervention** |
| --- | --- | --- | --- | --- | --- | --- | --- | --- | --- | --- | --- |
| Promoting high value inpatient care via a coaching model of structured, interdisciplinary team rounds | Artenstein et al. | 2015 | USA | Hospital | Care professional | General medicine/surgical ward | To assess, in descriptive fashion, the feasibility and the impact of incorporating an experienced physician into structured, interdisciplinary, ward rounds to coach the elements of high-value care. | Mixed Method | High-value care | Team-based care, Organize care around disease within setting | Active involvement in (see left) |
| Physician Experiences with High Value Care in Internal Medicine Residency: Mixed Methods Study of 2003–2013 Residency Graduates | Ryskina et al. | 2018 | USA | Hospital | Care professional | Internal medicine | To assess trends in US physician experiences with high value care during residency over the past decade. | Mixed Method | High-value care | Resource stewardship, SDM, Generic HVC or not specified, | Uncertain or not all professionals are actively involved in (see left) |
| What Do Doctors Think About Value-Based Healthcare? A Survey of Practicing Physicians in a Private Healthcare Provider in Brazil | Makdisse et al. | 2020 | Brazil | Hospital | Care professional | Not specified (all disciplines) | To capture awareness of value-based healthcare (VBHC). | Quantitative | Value-based care | Generic VBHC or not specified | Uncertain or not all professionals are actively involved in (see left) |
| Health professionals and students encounter multi-level barriers to implementing high-value osteoarthritis care: a multi-national study | Briggs et al. | 2019 | Australia, New Zealand and Canada | Physiotherapy Primary care | Care professional + student | Physiotherapy  Primary care | To explore barriers to high-value OA care delivery among clinicians and students. | Mixed Method | High-value care | Generic HVC or not specified | Uncertain or not all professionals are actively involved in (see left) |
| Experiences from implementing value-based healthcare at a Swedish University Hospital - a longitudinal interview | Nilsson, K et al. | 2017 | Sweden | Hospital | Care professional | Four different diagnostic groups | To explore how the representatives of four pilot project teams experienced implementing VBHC over a period of 2 years in four different groups of patients at a large Swedish University Hospital. | Qualitative | Value-based care | Organize care around disease within setting Team-based care Outcome measures Quality improvement Involve patient representatives | Active involvement in (see left) |
| The Role of the Pathologist in Population Health | Gross et al. | 2018 | USA | Medical laboratory | Care professional | Pathology Medical laboratory | To better understand the role pathologists can have in population health. | Qualitative | Value-based care | Population Health | Active involvement in (see left) |
| The Struggle Is Real: How Residents Learn to Provide High-Value, Cost-Conscious Care | Stammen et al. | 2019 | The Netherlands | Hospital, Primary care, Elderly care | Student | Dermatology Elderly care Family medicine Internal medicine Orthopaedics  Surgery | To offer insight into how residents learn to provide high-value, cost-conscious care in the workplace and how the postgraduate training environment influences this learning. | Qualitative | High-value, cost-conscious care | Generic HVCCC | Uncertain or not all professionals are actively involved in (see left) |
| Prospective Observational Study on High-Value Care Topics Discussed on Multidisciplinary Rounds | Beck et al. | 2018 | USA | Hospital | Care professional | General medicine Endocrinology Neurology Rheumatology Gastroenterology Nephrology Craniofacial | To explore the types of HVC discussions that take place at the bedside and who participates in those discussions. | Quantitative | High-value care | Discussing value in clinical encounter | Uncertain or not all professionals are actively involved in (see left) |
| Organization of outcome-based quality improvement in Dutch heart centres | Van Veghel et al. | 2019 | The Netherlands | Hospital | Care professional | Heart care | To evaluate the current state of outcome-based quality improvement within six of these Dutch heart centres | Mixed Method | Value-based care | Outcomes measures Quality Improvement | Active involvement in (see left) |
| Autonomy to health care professionals as a vehicle for value-based health care? Results of a quasi-experiment in hospital governance | Larsen et al. | 2018 | Denmark | Hospital | Care professional | General Medicine Ophthalmology Oral and Maxillofacial Surgery Orthopaedics Acute Medicine Ear, Nose and Throat Neurology Neurosurgery | To investigate the performance of eight hospital departments in the second largest region of Denmark that were delegated the authority to choose their own performance focus during a three-year test period from 2013-6. | Quantitative | Value-based care | Quality improvement (via delegated autonomy) | Active involvement in (see left) |
| The need to succeed - learning experiences resulting from the implementation of value-based healthcare | Nilsson, K et al. | 2018 | Sweden | Hospital | Care professional | Four different diagnostic groups | To explore learning experiences from the two first years of the implementation of VBHC at a large Swedish University Hospital. | Qualitative | Value-based care | Organize care around disease within setting Team-based care Outcome measures Quality improvement Involve patient representatives | Active involvement in (see left) |
| The strategic role of competency based medical education in health care reform: a case report from a small scale, resource limited, Caribbean setting | Busari & Duits | 2015 | Curacao | Diverse actors in care landscape | Diverse actors | Not specified | To investigate for the requirements needed to build a health care environment that is conducive for change and capable of facilitating the smooth migration of existent services into an effective and sustainable VBHC system. | Qualitative | Value-based care | Generic VBHC/not specified | Participant has not been exposed to VBHC (yet) |
| Governance Practices in an Era of Healthcare Transformation: Achieving a Successful Turnaround | Sondheim et al. | 2017 | USA | Hospital | Care professional | Emergency care | To examine a small community hospital’s successful transition from one emergency care center (ECC) physician group to another and the methods by which significant improvements in outcomes were achieved. | Mixed Method | Value-based care | Team-based care Outcome measures Quality improvement Accountability | Active involvement in (see left) |
| A realist evaluation of value-based care delivery in home care: The influence of actors, autonomy and accountability | Dainty et al. | 2018 | Canada | Home care | Care professional | Home care Community wound care services | To evaluate value-based initiative implementation. | Qualitative | Value-based care | Organize care around disease (across organizations) Team-based care Outcome measures Bundled payment | Active involvement in (see left) |
| How Do Attending Physicians Prepare Residents to Deliver High-Value, Cost-Conscious Care? | Stammen et al. | 2019 | The Netherlands | Hospital, Homecare, Elderly care | Care professional | Not specified | To understand how attending physicians, who have a central role in the workplace, prepare residents to provide HV3C. | Qualitative | High-value, cost-conscious care | Generic HVCCC or not specified | Uncertain or not all professionals are actively involved in (see left) |
| Association between Hospitalist Productivity Payments and High-Value Care Culture | Gupta et al. | 2018 | USA | Hospital | Care professional | Internal medicine | To evaluate if hospitalist reimbursement models are associated with high-value culture in university, community, and safety-net hospitals. | Quantitative | High-value care | Generic HVC, Resource stewardship | Uncertain or not all professionals are actively involved in (see left) |
| Medical Student Perceptions of Cost-Conscious Care in an Internal Medicine Clerkship: A Thematic Analysis | Tartagila et al. | 2015 | USA | Hospital | Student | Internal medicine | To examine medical students' perceptions of healthcare delivery as it relates to cost consciousness. | Qualitative | High-value care Cost-Conscious Care | Generic HVCCC, Resource stewardship | Uncertain or not all professionals are actively involved in (see left) |
| Value-based healthcare as a trigger for improvement initiatives | Nilsson, K. et al. | 2017 | Sweden | Hospital | Care professional | Four different diagnostic groups | To explores four pilot teams’ experiences of improvements resulting from the implementation of value-based healthcare (VBHC) at a Swedish University Hospital. The aim of this study is to gain a deeper understanding of VBHC when used as a management strategy to improve patients’ health outcomes. | Qualitative | Value-based care | Organize care around disease within setting Team-based care Outcome measures Quality improvement Evidence & tailor Benchmark | Active involvement in (see left) |
| Exploring the applicability of the pregnancy and childbirth outcome set: A mixed methods study | Laureij et al. | 2020 | The Netherlands | Hospital, Primary care, Maternity care, Midwifery practice | Care professional | Perinatal care | To assess views on the PCB set. | Mixed Method | Value-based care | Outcome measures Collaborate in chain, Involve patient representatives | Active involvement in (see left) |
| Imprinting on Clinical Rotations: Multisite Survey of High and Low-Value Medical Student Behaviors and Relationship with Healthcare Intensity | Hunderfund et al. | 2019 | USA | Hospital | Student | Not specified | To explore potential imprinting on clinical rotations by (a) describing high- and low-value behaviors among medical students and (b) examining relationships with regional healthcare intensity (HCI). | Quantitative | High-value care Low-value care | Resource stewardship | Uncertain or not all professionals are actively involved in (see left) |
| Critical Lessons From High-Value Oncology Practices | Blayney et al. | 2017 | USA | Ambulatory care | Care professional | Oncology | To assess oncology practice attributes distinguishing “high value” that may be tested and adopted by others to produce similar results. | Qualitative | High-value care | Generic VBHC or not specified | Active involvement in (see left) |
| Qualifications and Competencies for Population Health Management Positions: A Content Analysis of Job Postings | Meyer | 2017 | USA | Not applicable | Not applicable (about all care professionals) | Not applicable | To analyze the types of population health management positions for which health care organizations are hiring, including qualifications and competencies required for these positions. | Qualitative | Value-based care | Population health | Not applicable |
| Multiyear Performance Trends Analysis of Primary Care Practices Demonstrating Patient-Centered Medical Home Transformation: An Observation of Quality Improvement Indicators among Outpatient Clinics | Pereira et al. | 2019 | USA | Primary care | Not applicable (about all care professionals) | Primary care | To describe national trends in National Committee for Quality Assurance PCMH recognition for more than 23 000 primary care practices across the United States from 2008 to 2017. | Quantitative | Value-based care | Patient-Centered Medical Home (PCMH) | Active involvement in (see left) |
| Understanding Factors Influencing Quality Improvement Capacity Among Ambulatory Care Practices Across the MidSouth Region: An Exploratory Qualitative Study | Varley et al. | 2020 | USA | Ambulatory care | Care professional | Ambulatory care | To explore factors influencing QI capacity among ambulatory care practices in the MidSouth Practice Transformation Network. | Qualitative | Value-based care | Quality improvement | Uncertain or not all professionals are actively involved in (see left) |
| Findings from FMAHealth's Bright Spots in Practice Transformation Project | Robinson et al. | 2019 | USA | Primary care | Care professional | Family physician | To identify drivers of transformation to value-based care and ways of working with drivers to mitigate potential barriers, and to determine relationships between practice transformation and joy of practice. | Qualitative | Value-based care | Practise transformation to value-based model, seemingly including:  Organize care around disease within setting Team-based care Outcome measures Engaged leadership | Active involvement in (see left) |
| A value-based taxonomy of improvement approaches in healthcare | Collden et al. | 2017 | Sweden | Hospital, outpatient care | Care professional | Psychiatry | To construct a taxonomy that supports management of parallel IA’s in healthcare. | Qualitative | Value-based care | Overall VBHC/ not specified | Active involvement in (see left) |
| Perceptions of Ambulatory Workflow Changes in an Academic Primary Care Setting | Hanak et al. | 2017 | USA | Ambulatory care, Primary care | Care professional | Ambulatory care, Primary care | To evaluate various components of a CMA (certified medical assistants (CMAs)) workflow in adult primary care practices within an academic medical center. | Quantitative | Value-based care High-value care | Workflow redesign | Active involvement in (see left) |
| High-Value Care Culture Among the Future Physician Workforce in Internal Medicine | Gupta et al. | 2019 | USA | Hospital | Student | Internal medicine | To evaluate whether trainees’ exposure to a high-value care culture differed based on type of health system in which they trained. | Quantitative | High-value care | Generic VBHC or not specified | Uncertain or not all professionals are actively involved in (see left) |
| Redefining value: a discourse analysis on value-based health care | Steinmann et al. | 2020 | The Netherlands | Diverse actors in care landscape | Diverse actors | Not applicable | To map the ambiguity surrounding VBHC. | Qualitative | Value-based care | Generic VBHC or not specified | Uncertain or not all professionals are actively involved in (see left) |
| Surgeons’ Preoperative Work Burden Has Increased Before Total Joint Arthroplasty: A Survey of AAHKS Members | Grosso et al. | 2020 | USA | Hospital | Care professional | Orthopaedics | To quantify the work burden associated with preoperative TJA care. | Quantitative | Value-based care | Organize care around disease within setting Care Pathway | Active involvement in (see left) |
| High-Value, Cost-Conscious Care Attitudes in the Graduate Medical Education Learning Environment: Various Stakeholder Attitudes That Residents Misjudge | Mordang et al. | 2020 | The Netherlands | Hospital | Care professional, student | Not specified | To measure stakeholders’ HVCCC attitudes in residents’ learning environment, compare these with resident perceptions of their attitudes, and identify factors associated with attitudinal differences among each stakeholder group. | Quantitative | High-value, cost-conscious care | Generic HVCCC or not specified | Uncertain or not all professionals are actively involved in (see left) |
| Choosing Wisely in Critical Care: A National Survey of Critical Care Nurses | Wiencek et al. | 2019 | USA | Hospital | Care professional | Acute and ICU clinical practice | To assess nurses’ reports of the use of Choosing Wisely recommendations in critical care settings. | Mixed Method | High-value care | Resource stewardship Guidelines | Uncertain or not all professionals are actively involved in (see left) |
| Qualitative Study to Understand Pediatric Hospitalists and Emergency Medicine Physicians' Perspectives of Clinical Pathways | O'hara et al. | 2020 | USA | Hospital | Care professional | Pediatric (emergency) medicine | To examine pediatric hospital medicine (PHM) and pediatric emergency medicine (PEM) physician perspectives of clinical pathways. | Qualitative | High-value care | Pathway (re)design | Active involvement in (see left) |
| Readiness of hospital-based internists to embrace and discuss high-value care with patients and family members: a single-centre cross-sectional survey study | Brandt Vegas et al. | 2015 | Canada | Hospital | Care professional | Internal medicine | To determine how ready practicing internists were to embrace and openly address high-value care during conversations with patients or their families. | Quantitative | High-value care | Discussing value in the clinical encounter Resource stewardship | Uncertain or not all professionals are actively involved in (see left) |
| High-value care for older adults with complex care needs: Leveraging nurses as innovators | Demiris et al. | 2020 | USA | Diverse actors in care landscape | Care professional | Gerontology | To introduce a series of recommendations for leveraging nurses to generate innovative tools and solutions for the delivery of value-based care for older adults living with complex health and social needs and their families. | Qualitative | Value-based care High-value care | Leadership in generic VBHC/ not specified | Uncertain or not all professionals are actively involved in (see left) |
| The use of PROMs and shared decision-making in medical encounters with patients: An opportunity to deliver value-based health care to patients | Damman et al. | 2019 | The Netherlands | Hospital | Care professional | Neurology Oncology Mental health care | To describe approaches and lessons learned in the fields of SDM and VBHC implementation that converge in using PROMs in medical encounters. | Qualitative | Value-based care | Outcome measures SDM | Active involvement in (see left) |
| Leadership skills essential in the value-based care era | Cornell | 2020 | USA | ACO | Care professional (Executive leader) | Not specified | What primary executive leadership skills are essential in the VBCD era. | Qualitative | Value-based care | Leadership in generic VBHC/not specified | Active involvement in (see left) |
| Nurse professional competence (NPC) assessed among newly graduated nurses in higher educational institutions in Europe | Nilsson, J. et al. | 2019 | Europe (11 institutions) | Not specified | Student | Not specified | To assess and compare nursing education and self-reported professional competence among nursing students graduating with a bachelor’s degree from higher education institutions in Europe. | Quantitative | Value-based care | Generic VBHC or not specified | Uncertain or not all professionals are actively involved in (see left) |
| The Nurse Professional Competence (NPC) Scale: A tool that can be used in national and international assessments of nursing education programmes | Gardulf et al. | 2019 | Sweden | Not specified | Student | Not specified | To investigate whether the Nurse Professional Competence (NPC) Scale could serve as a tool to measure and detect possible differences between universities/university colleges regarding nursing students’ self-reported competence. | Quantitative | Value-based care | Generic VBHC or not specified | Uncertain or not all professionals are actively involved in (see left) |
| Understanding value-based healthcare – an interview study with project team members at a Swedish university hospital | Erichsen Andersson et al. | 2015 | Sweden | Hospital | Care professional | Psychiatry Oncology Orthopaedics Heart care | To explore how representatives from four project teams understand the concept of value-based Healthcare. | Qualitative | Value-based care | Organize care around disease within setting Team-based care Outcome measures Quality Improvement, Involve patient representatives Benchmark | Active involvement in (see left) |
| Leading implementation of the management innovation value-based healthcare at a Swedish University Hospital | Nilsson & Sandoff | 2017 | Sweden | Hospital | Care professional | Psychiatry Oncology Orthopaedics Heart care | To explore medical and care staff’s experiences of leading the implementation of the management innovation, named value-based healthcare (VBHC). | Qualitative | Value-based care | Outcome measures Quality improvement | Active involvement in (see left) |
| Moving Organizational Culture from Volume to Value: A Qualitative Analysis of Private Sector Accountable Care Organization Development | McAlearnly et al. | 2018 | USA | ACO | Care professional | Not specified | To explore the cultural change necessary for shifting from volume to value in ACO's. | Qualitative | Value-based care | Accountable care  Population health | Active involvement in (see left) |
| Value-based healthcare translated: a complementary view of implementation | Colldén & Hellström | 2018 | Sweden | Hospital | Care professional | Psychiatry | To investigate how a translation theory perspective can inform the Consolidated Framework of Implementation Research (CFIR) to increase understanding of the complex process of putting MIs into practice. | Qualitative | Value-based care | Outcome measures Value discussions  (Benchmark), Organize care around disease within setting | Active involvement in (see left) |
| Understanding value in oral health: the oral health value-based care symposium | Boynes et al. | 2020 | USA | Oral health | Diverse actors | Oral health | To develop a gap analysis and capture insights into professional readiness for value-based care design. | Mixed Method | Value-based care | Generic VBHC or not specified | Uncertain or not all professionals are actively involved in (see left) |
| U.S. Internal Medicine Residents’ Knowledge and Practice of High-Value Care: A National Survey | Ryskina et al. | 2015 | USA | Hospital | Student | Internal medicine | To determine U.S. internal medicine (IM) residents’ knowledge of, attitudes toward, and self-reported practice of high-value care (HVC), or care that balances the benefits, harms, and costs of tests and treatments. | Quantitative | High-value care | Generic HVC or not specified | Uncertain or not all professionals are actively involved in (see left) |
