# Supplementary material for: Value-Based Healthcare From the Perspective of the Healthcare Professional: A Systematic Literature Review
Source: Front Public Health. 2022 Jan 13;9:800702. doi: 10.3389/fpubh.2021.800702 (PMC8792751; doi:10.3389/fpubh.2021.800702)
Supplement: Supplementary file 4 [file Data_Sheet_4.docx]

Findings on VBHC specific elements

This complementary file elaborates on the findings stated in the main text regarding the ‘VBHC specific’ elements. These elements comprised the ‘professional’, the ‘job’ of pursuing value in care and the ‘environment’ as depicted in the left column of Figure 1.


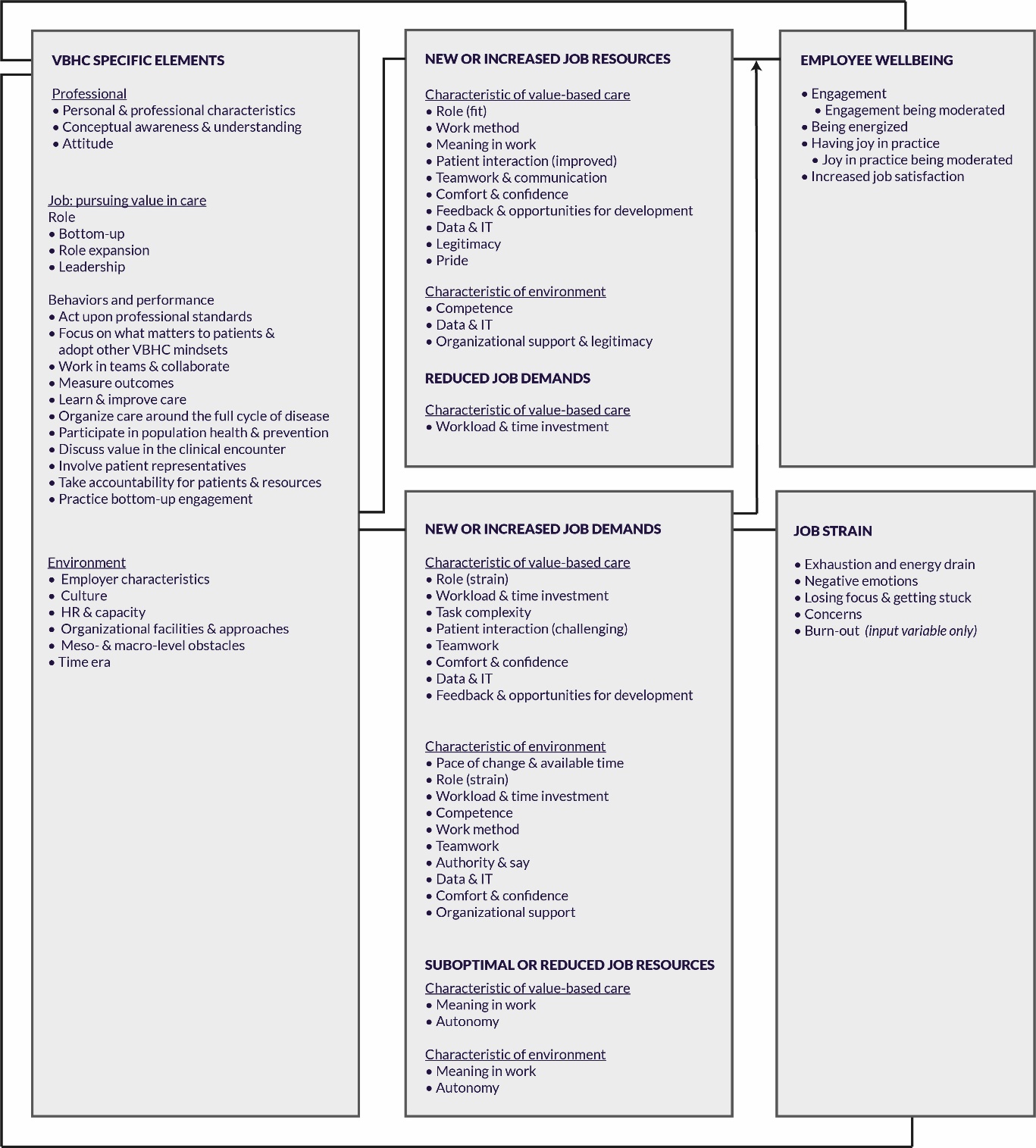


Figure 1. Result from thematic analysis using the modified J D-R model

# The professional

The element ‘professional’ covers the ‘personal & professional characteristics’, ‘conceptual awareness & understanding’ and ‘attitudes towards VBHC’ of healthcare professionals. Table 1 provides an overview.

## Personal & professional characteristics

Studies reported on an association between age and gender, as personal characteristics, and VBHC awareness (1), HVCCC scores (2), and attitudes towards VBHC (3). Other personal characteristics in VBHC mentioned were pro-activeness (4), criticalness (5) and attributing importance to new technologies and public disclosure of patient satisfaction surveys (1). Outcomes were studied in relation to, and compared between, employee characteristics such as job position (1–3,6,7).

## Conceptual awareness & understanding

Professionals’ awareness was studied in relation to VBHC (1), choosing wisely recommendations (7), Shared Decision Making (SDM) (8) and VBHC implementation (9). Studies investigated how professionals perceived value and VBHC, also in relation to other management innovation tools (10), and showed that understanding and interpretation was not uniform and sometimes involved prioritization of patient outcomes or costs (1,5,16,6,9–15). Rapid pace of VBHC implementation was suggested to negatively impact the development of understanding (9,16).

## Attitudes towards VBHC

Mixed attitudes towards VBHC were reported. On the positive side, VBHC was received with excitement and enthusiasm, convincement and with suggested readiness (1,2,17–21,3,5,9,11–14,16). VBHC was perceived positively, as commendable and to trigger hope. Although VBHC was assumed to connect to professionals intrinsic motivation (11), none of the included studies investigated motivation. Participants expressed relevance of outcome measures (1,17), which made them accept the necessary registration (12). Some professionals reported on their motives for integrating costs in care delivery (5,19). Participants also felt urgency to improve care in case of low outcome scores (18), and expressed interest to participate in improvements (2) and to receive feedback on their value-based behaviors (19).

However, negative attitudes were also reported (3,5,16,19–23,7–12,14,15), for example in the form of critique (11), drawbacks (3), ignorance (9) and resistance (5,7,15,16). Possible explanations for these negative attitudes were prior experiences with time-consuming coding and meaningless outcomes (14), tiredness of the cost-focus that had been existing for long (16), residents’ short-term involvement (5) and other root-cause problems (16). Professionals seemed reluctant to consider costs (5,10,19,22) and to discuss costs openly (19,21), although positive attitudes towards cost incorporation were mentioned as well (19). Furthermore, professionals reported on possible misconceptions in relation to: the need of SDM and the necessary time for SDM (8), the possibility to benchmark (12) their power in VBHC (23).

Table 1. Overview and illustrative quotes about the professional in VBHC

| **Professional** |  | **Studies** | **Exemplifying quote** |
| --- | --- | --- | --- |
| Personal & professional characteristics |  | (1–7) | “Among residents, male gender and [...] were associated with more favorable attitudes toward high- value care (β = 0.09, 95% CI = 0.03, 0.16, p = 0.006; p = 0.006). However, male residents also endorsed more potential drawbacks (β = 0.13, 95% CI = 0.04, 0.21, p = 0.004), as did younger residents (β = 0.02, 95% CI = 0.03, 0.00, p = 0.01)” (3) |
| Awareness & understanding | Awareness | (1,7–9) | “Twenty-seven percent of physicians rated their awareness of VBHC as high or very high” (1) |
|  | Conceptual understanding and factors impacting development of understanding | (1,5,16,6,9–15) | “Four discourses on VBHC:] Firstly, there is what we have labeled a Patient Empowerment discourse (PEMP), in which VBHC is chiefly por- trayed as a framework for strengthening the position of patients regarding their medical decisions. Secondly, we have identified a Governance discourse (GOV) in which VBHC is primarily construed as a mechanism to steer and regulate care providers toward value for patients. Third, there is a Professionalism discourse (PROF), in which VBHC is predominantly construed as a methodology for the organization and improvement of health care delivery. Fourthly, we have identified a Critique discourse (CRI), which is characterized by a specific form of critique of VBHC, particularly its emphasis on measurement and standardization” (24) |
| Attitude towards VBHC | Positive | (1,2,17–21,3,5,9,11–14,16) | “In sum, individuals with different points of view could all attach hope to the new fuzzy concept VBHC” (14) |
|  | Negative | (3,5,16,19–23,7–12,14,15) | “They found it [HV3C] hard, sometimes even undesirable, to translate into practice. […] One of the residents who participated in the focus groups felt very strongly that it was incumbent upon residents to do everything in their power to help the patient, however costly” (5) |

# The job: pursuing value in care

Studies described behaviors of professionals in VBHC and their performance. Studies suggested that VBHC is a bottom-up initiative that expanded roles, established new roles and called for leadership. Although none of the included studies provided overview of all the behaviors in VBHC, thematic analysis revealed ten behaviors. These behaviors, which build upon professionals acting upon their professional standards (25), are interconnected and mutually reinforcing. More information about professionals’ roles and the VBHC behaviors are detailed below. An overview is provided in Figure 2 and Table 2.

## Bottom-up role

Studies reported on VBHC as bottom-up initiatives (7,11–13,16,18,20,26). Top-down approaches were considered not appropriate. One study investigated the delegation of authority to professionals and found a neutral effect on quality improvements (27).

## Role expansion & new roles

VBHC was associated with role expansion and new roles (4,16,33,17,18,26,28–32). For example, roles were expanded to include discussion of patient reported outcomes (17,29), coaching of peers (28), championship (4) and research-skilled professionals were assumed to guarantee critical thinking (16). Medical assistants (32,33) and nurses (26,30,31) saw or suggested expansion of their tasks and function, such as the establishment of contact nurse function. Professionals from less obvious disciplines to participate in VBHC, such as pathologists and professionals in dental care, felt the urge to express their relevance (4,34). Studies suggested that roles in VBHC still need to be formalized (17,18), while one of these studies also indicated that professionals prefer no mandates (18).

## Leadership characteristics

Engaged leadership was described necessary to involve and engage staff (9,13,20,32,35), while this also depended on the attitude and autonomy of the staff (20). Important leadership characteristics and competences included approachability, having vision, perseverance and positivity. Nurses (31) and pathologists (4), were two professions described to potentially take up leadership roles in the future.

## VBHC behaviors

Thematic analysis revealed ten specific behaviors that professionals pursued in VBHC, next to acting upon their professional standards. These interconnected and mutually reinforcing behaviors, as visualized in Figure 2, are to 1) focus on what matters to patients & adopt other VBHC mindsets 2) measure outcomes, 3) learn & improve care, 4) organize care around the full cycle of disease, 5) participate in population health & prevention, 6) discuss value in the clinical encounter, 7) involve patient representatives, 8) take accountability for patients & resources, 9) practice bottom-up engagement and above all: 10) work in teams.


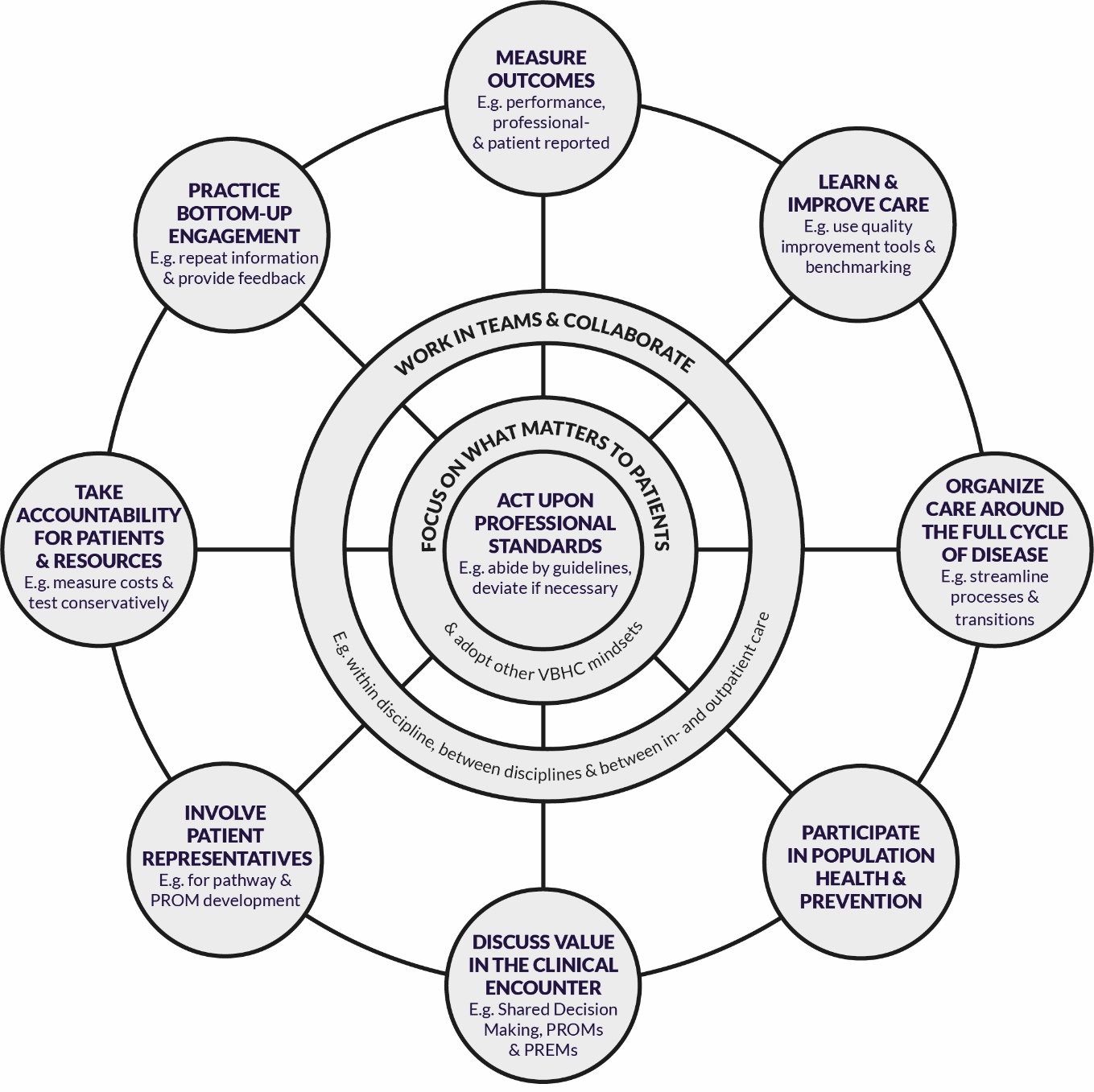


Figure 2. Professionals’ behaviors to pursue value in care

### Focus on what matters to patients & adopt other VBHC mindsets

Ten studies reported on professionals starting to think differently and adopt new mindsets (4,7–12,16,26,28). A challenge professionals faced was to truly focus on what matters to patients and take unexpected answers as basis since risks existed that professionals were (unintentionally) driven by their own values (8–12,16,26,28). Especially for benchmarking an open mindset was considered important. Professionals were suggested to view benchmarking as an opportunity for learning rather than a moment to explain away their lower scores (12). This open mindset was also considered critical to address each other on outcomes (17) and start collaboration with less obvious partners (4). Furthermore, professionals adjusted their expectation of quick results and now viewed VBHC as ‘never-ending’ (16). Other changes in mindset were to take research as basis for changes (16) and to view conservative testing as the new norm (7).

### Measure outcomes

Various actions in relation to outcome measures were described (9,12,14,16–18,20,25,26,36). These included establishment and use of performance metrics, professional-reported outcomes and patient-reported outcome measures (PROMs). Amongst others, professionals mapped current processes and measurements and investigated what needed to be measured and how this could be achieved (9,12,14,16,20). Routines and technology to facilitate data capture were revised or developed (14,16,26) and finally data was captured (9,16–18,36).

### Learn & improve care

Amongst others by discussing outcome data, professionals identified and made improvements (4,7,32,37,38,9,11,12,16,18,20,25,26). This process was facilitated by use of Quality Improvement (QI) methods such as LEAN (32) and Choosing Wisely recommendations (7). Professional started by identifying the root cause (12,26). Improvements were combined (16) and if needed current practices were discarded (4,12,14,37). Professionals were also suggested to aim for demonstrating best practices (25), next to their engagement in benchmarking (14,18,26). QI was used as a criterion for ‘medical home’ assessment, which showed repetitive underperformance in various cases (38).

### Organize care around the full cycle of disease

Professionals organized care around the full cycle of disease (13,16,38–40,26–28,30,32–34,36). This was done based on measurements (26) and pathways (40). Care was planned in collaboration with patients at admission and with staff working on the whole care process (26). Moreover, professionals proactively addressed and streamlined care processes and transitions (28,32,34) by internal cooperation with other departments (16) and by cooperation between in-and outpatient care (13,16). For example, professionals explicitly discussed the most efficient testing route to patient goals (30) and provided proactive support (30) and preoperative services (39). Although improved care planning was mentioned (27,36,38), care professionals had different perceptions about professionals’ adherence to new workflows (33). Improvement potential was identified for referral tracking, for follow-up and patient-centered care collaboration between clinical and nonclinical team members and between primary care provider and specialty care providers (38).

### Participate in population health & prevention

Care coordination was also considered essential when professionals engaged in population health and prevention (4,16,32,34,38), namely to counteract possible adverse effects related to use of care services and hence safeguard sustainability (32). Although prevention was considered important in VBHC (34), studies did not report on specific preventative behaviors.

### Discuss value in the clinical encounter

In VBHC professionals with direct patient contact discussed value in the clinical encounter (5,7,8,12,17,21,29,30,41,42). Patients viewed it was the role of healthcare professionals to discuss PROMs, especially the more sensitive topics (17). At the same time many professionals preferred to discuss value implicitly (21). One observation-based study reported that in 30 per cent of patient encounters a value-related topic was discussed (28). Most occurring VBHC topics discussed were avoidance of a low-value tests and the tailoring of care plans. Other studies reported that professionals asked for treatment experience (12) and discussed treatment planning (7,30) and medication (5). There was variety in the extent costs were discussed (41,42) and SDM was applied (8).

### Involve patient representatives

Professionals took efforts to involve patient representatives (8,9,12,16,26). Involving patient representatives was considered important for PROM development (8). Moreover, involvement of patient representatives strengthened the focus on value for patients (9,16) and raised awareness of improvement potential (26). Involvement of patient representatives was considered to ‘add something’ (12) while professionals were also aware of the necessary delicacy and preparation that involvement of patient representatives demanded (9).

### Take accountability for patients and resources

VBHC asked professionals to take accountability (13,25,36), especially for the care of patients and use of resources (2,5,30,33,36,37,41,42,7,9,12,13,17,19,22,25). Professionals took accountability for resources by attempting to measure costs (9,12), studying cost theory (19), prescribing generic medication and reviewing medicine (22,33) discussing costs of care (25,41), weighing costs in decision-making (2,37,42) and discussing conservative testing (30). However, improvement potential was identified (7). Due to limited sense of resource-stewardship in junior residents (41), one study described junior residents as potential delivers of wasteful care and hence in need of guidance or limits in their autonomy (19).

### Practice bottom-up engagement

Staff involvement was considered essential (7,9,12,14,16,18,20,26). Efforts were taken to inform and engage staff pedagogically using informal dialogues with repeated information (9,20) and by fostering their confidence (20). Also, amongst others, professionals shared examples of good practices (16), provided feedback (20), used prompts (7) and made VBHC appear locally invented (14). Resistance was overcome by discarding existing measures when possible (12,14). However, reflection learned that staff engagement was not yet optimal and may need other approaches (18,26).

### Work together & collaborate

Studies reported on deployment of team-based models in VBHC and increased collaboration (4,8,12,20,25,28,32,34,43). Professionals collaborated during VBHC implementation, improvement work, when preparing patient consultations and during patient care itself. Various actors were mentioned as a member of the multidisciplinary team, such as experienced physicians, sub-specialists, medical assistants, care coordinators, nurse managers, social workers, pharmacists, respiratory therapists, and bedside nurses.

Table 2. Overview and illustrative quotes on the job of pursuing value in care

| **Job: pursuing value in care** |  | **Studies** | **Exemplifying quote** |
| --- | --- | --- | --- |
| Role | Bottom-up | (7,11–13,16,18,20,26) | “Outcome performance and improvement is partially discussed within specialties (the doctor’s unit ‘cardiothoracic surgery’), partially appropriated by initiatives from individual physicians and partially in project teams. […].physicians taking initiative either individually or within project teams generally do not enjoy a formal mandate within the organization” (18) |
|  | Role expansion & new roles | (4,16,33,17,18,26,28–32) | “As the implementation work preceded, the contact nurse’s function became more established. After two years, continuous measurements showed that 90 per cent of the patients were appointed a contact nurse (IP17) ” (26) |
|  | Leadership | (9,13,20,32,35) | [Study on leadership skills essential in the value-based care era] (35) |
| Behavior and performance | Act upon professional standards | (25) | “They should act in accordance with the responsibilities as defined by their respective professional standards, The medical specialist is expected to provide good health care services that meet acceptable standards i.e. are safe, effective, patient centered, delivered timely and commensurate with the patient’s real needs. The medical specialist abides by the professional guidelines/protocols that apply to him, and may deviate from these, if and when necessary, [continued]” (25) |
|  | Focus on what matters to patients & adopt other VBHC mindsets | (4,7–12,16,26,28) | “Over time, participants questioned their own thinking and said they had had to learn a new way of thinking, integrating the patients’ perspective in their more traditional profession-based thinking” (9) |
|  | Measure outcomes | (9,12,14,16–18,20,25,26,36) | “To solve these problems, they established a new coding system and a new working routine ” (26) |
|  | Learn & improve care | (4,7,32,37,38,9,11,12,16,18,20,25,26) | “The participants found that measuring different variables contributed to the possibility of identifying not only what they needed to do better, but also what they did wrong. Detecting divergences in the scorecards led to improvements in care processes as well as the development of new procedures” (16) |
|  | Organize care around the full cycle of disease | (13,16,38–40,26–28,30,32–34,36) | “Proactive support for patients during predictably stressful” (30) |
|  | Participate in population health & prevention | (4,16,32,34,38) | “Key characteristics associated with the best practices for the current state OHVBC [oral health value based care] were discussed among the expert participants, including prevention innovation, expanded workforce, and health outcome measures for prevention” (34) |
|  | Discuss value in the clinical encounter | (5,7,8,12,17,21,29,30,41,42) | “Overall, 29% of all patient encounters (191 of 660; 95% CI: 26%–33%) included at least 1 observed HVC discussion from the 10 potential topics ” (29) |
|  | Involve patient representatives | (8,9,12,16,26) | “Importance of listening to patient representatives. […]. involves putting questions to patients” (12) |
|  | Take accountability for patients & resources | (2,5,30,33,36,37,41,42,7,9,12,13,17,19,22,25) | “45% re-ported weighing costs in clinical decision making” (37) |
|  | Practice bottom-up engagement | (7,9,12,14,16,18,20,26) | “Therefore, in order to forestall organizational resistance and gain acceptance among professionals, the project group first focused on reducing measurements that professionals perceived as meaning- less and time consuming” (14) |
|  | Work in teams & collaborate | (4,8,12,20,25,28,32,34,43) | “All practices interviewed had moved to team-based care and indicated it was a primary driver of practice transformation” (32) |

# The Environment

Analysis identified several characteristics of the environment that were measured or discussed from the perspective of the healthcare professional. These characteristics, as shown in Table 3, related to culture, HR & capacity, organizational facilities and approaches, other meso- and macro-level obstacles and the time era.

## Employer characteristics

Employer characteristics such as type of hospital (2,37), region (3,38,44), health-care intensity (41) and number of clinicians (38) were related to, amongst others, self-reported knowledge, perceived barriers, behaviors and performance in VBHC (2,3,5,29,30,37,38,41,42,44).

## Culture

Participants mentioned the need for cultural change (7,12,16,17), and more specifically cultures that are transparent, blame-free, self-critical and focus on improvement as well as cultures where professionals address each other on outcomes (2,11,12,17,18,32,34,37).

## HR & capacity

Professionals reported on staffing constraints, both for professionals and supportive personnel (6,9,23,28). Although staff stability was considered important (16,23,32), concern was expressed about nurses trained in VBHC leaving for promotions at other practices (32). Besides numerous studies reporting on deployment of third-party services, consultants, data analysis and econometrists, e.g. (9,14,43,16,18,30–34,39), studies also reported on specific staffing needs (4,9,20,34,45) such as current open positions in population health functions (45).

## Organizational facilities & approaches

Thematic analysis revealed various latent needs and desires of professionals. These related to provision of dedicated time for VBHC (18,23,32) as lack of time was considered a possible barrier (23), the adoption of a step-by-step approach (14,17,20,34,43) and supportive IT and organizational commitment and investment in VBHC (4,11,34,40,12,13,16,17,19,23,31,32), with special attention to engaged leadership (9,13,14,18,32).

## Other meso-, and macro-level obstacles

Professionals also identified organizational-level and system-level challenges that formed obstacles to VBHC but were not explicitly labeled as a demand. These obstacles included patients’ limited access to care (6) and lack of understanding of VBHC (13), organizational obstacles (14,16) such as conflicting interests (6), and lack of commitment and messaging from ministry (43).

## Time era

One study addressed the time era by stating that Covid19 was considered to have a progressive impact on VBHC (34).

Table 3. Overview and illustrative quotes on reported environment-related aspects in VBHC

| **Environment** |  | **Studies** | **Exemplifying quote** |
| --- | --- | --- | --- |
| Employer characteristics |  | (2,3,5,29,30,37,38,41,42,44) | “Hospital region was significantly associated with faculty’s attitudes toward high-value care (p = 0.002) and cost incorporation (p = 0.004), but not with their beliefs about potential drawbacks” (3) |
| Culture | Culture change | (7,12,16,17) | “a culture change is needed in order to create an environment in which it is normal to address each other on outcomes” (17) |
|  | Assessment and need of specific culture | (2,11,12,17,18,32,34,37) | “A culture exists to openly discuss outcomes within each specialty, but less openness exists to discuss outcomes in a multidisciplinary setting” (18) |
| HR & capacity | Staffing constraints | (6,9,23,28) | “Limitations with the service environment, including inadequate human resources” (6) |
|  | Staff composition | e.g. (9,14,43,16,18,30–34,39) | “Care Team Functions at the Highest Level of Competence and License. The use of experienced oncology nurses and other nononcologist care providers was another often-mentioned attribute [of high-value practices] ” (43) |
|  | Staff stability | (16,23,32) | “Once trained at the highest scope of their licenses, they found medical assistants and registered nurses may leave for promotions as office managers and care managers, respectively, at other local practices” (32) |
|  | Need for certain staff | (4,9,20,34,45) | “Qualifications and competencies for population health management positions” (45) |
| Organizational facilities and approaches | Provide dedicated time | (18,23,32) | “Others stated that dedicated time and […] were key activities to sustaining improvements” (32) |
|  | Adopt a focused, step-by-step approach | (14,17,20,34,43) | “The anchoring process was facilitated by implementing changes in small steps” (17) |
|  | Overall supportive environment (policy, IT etcetera) | (4,11,34,40,12,13,16,17,19,23,31,32) | “Additionally, they mentioned their need for supportive data on their own behavior and the opportunity to compare their data with the data of colleagues” (19) |
|  | Engaged leadership | (9,13,14,18,32) | “Transformation did not happen by chance; it was initiated by a leader who recognized the importance of value-based care and was sustained through engaged leadership” (32) |
| Other meso-, and macro-level obstacles |  | (6,13,14,16,43) | “Perceived conflicts between industry sector interests and what might be best for the patient were also highlighted by some [as barrier] ” (6) |
| Time era |  | (34) | “80 percent of respondents believed that the COVID-19 pandemic would have a progressive impact on OHVBC [oral health value based care]” (34) |

**References**

1. Makdisse M, Ramos P, Malheiro D, Felix M, Cypriano A, Soares J, et al. What Do Doctors Think About Value-Based Healthcare? A Survey of Practicing Physicians in a Private Healthcare Provider in Brazil. Value Heal Reg Issues. 2020;23(C):25–9. doi: 10.1016/j.vhri.2019.10.003

2. Gupta R, Steers N, Moriates C, Wali S, Braddock CH, Ong M. High-Value Care Culture Among the Future Physician Workforce in Internal Medicine. Acad Med. 2019;94(9):1347–54. doi: 10.1097/acm.0000000000002619

3. Mordang SBR, Leep Hunderfund AN, Smeenk FWJM, Stassen LPS, Könings KD. High-Value, Cost-Conscious Care Attitudes in the Graduate Medical Education Learning Environment: Various Stakeholder Attitudes That Residents Misjudge. J Gen Intern Med. 2020; doi: 10.1007/s11606-020-06261-8

4. Gross DJ, Kennedy M, Kothari T, Scamurra DO, Wilkerson ML, Crawford JM, et al. The Role of the Pathologist in Population Health. Arch Pathol Lab Med. 2019;143:610–20. doi: 10.5858/arpa.2018-0223-CP

5. Stammen L, Slootweg I, Stalmeijer R, Janssen L, Stassen L, Scheele F, et al. The Struggle Is Real: How Residents Learn to Provide High-Value, Cost-Conscious Care. Teach Learn Med. 2019;31:402–11. doi: 10.1080/10401334.2019.1583566

6. Briggs AM, Houlding E, Hinman RS, Desmond LA, Bennell KL, Darlow B, et al. Health professionals and students encounter multi-level barriers to implementing high-value osteoarthritis care: a multi-national study. Osteoarthr Cartil. 2019;27:788–804. doi: 10.1016/j.joca.2018.12.024

7. Wiencek CA, Kleinpell R, Moss M, Sessler CN. Choosing Wisely in Critical Care: A National Survey of Critical Care Nurses. Am J Crit Care. 2019;28:434–40. doi: 10.4037/ajcc2019241

8. Damman OC, Jani A, Jong BA, Becker A, Metz MJ, Bruijne MC, et al. The use of PROMs and shared decision‐making in medical encounters with patients: An opportunity to deliver value‐based health care to patients. J EVAL CLIN Pr. 2020;26:524–40. doi: 10.1111/jep.13321

9. Nilsson K, Bååthe F, Erichsen Andersson A, Sandoff M. The need to succeed – learning experiences resulting from the implementation of value-based healthcare. Leadersh Heal Serv. 2018;31(1):2–16. doi: 10.1108/LHS-08-2016-0039

10. Colldén C, Gremyr I, Hellström A, Sporraeus D. A value-based taxonomy of improvement approaches in healthcare. J Heal Organ Manag. 2017;31(4):445–58. doi: 10.1108/JHOM-08-2016-0162

11. Steinmann G, van de Bovenkamp H, de Bont A, Delnoij D. Redefining value: a discourse analysis on value-based health care. BMC Heal Serv Res. 2020;20:862. doi: 10.1186/s12913-020-05614-7

12. Erichsen Andersson A, Bååthe F, Wikström E, Nilsson K. Understanding value-based healthcare – an interview study with project team members at a Swedish university hospital. J Hosp Adm. 2015;4(4):64. doi: 10.5430/jha.v4n4p64

13. McAlearney AS, Walker DM, Hefner JL. Moving Organizational Culture from Volume to Value: A Qualitative Analysis of Private Sector Accountable Care Organization Development. Health Serv Res. 2018;53(6):4767–88. doi: 10.1111/1475-6773.13012

14. Colldén C, Hellström A. Value-based healthcare translated: A complementary view of implementation. BMC Health Serv Res. 2018;18(1):1–11. doi: 10.1186/s12913-018-3488-9

15. Tartaglia KM, Kman N, Ledford C. Medical Student Perceptions of Cost-Conscious Care in an Internal Medicine Clerkship: A Thematic Analysis. J Gen Intern Med. 2015;30:1491–6. doi: 10.1007/s11606-015-3324-4

16. Nilsson K, Bååthe F, Andersson AE, Wikström E, Sandoff M. Experiences from implementing value-based healthcare at a Swedish University Hospital - an longitudinal interview study. BMC Health Serv Res. 2017;17(1):169. doi: 10.1186/s12913-017-2104-8

17. Laureij LT, Been J V, Lugtenberg M, Ernst-Smelt HE, Franx A, Hazelzet JA, et al. Exploring the applicability of the pregnancy and childbirth outcome set: A mixed methods study. Patient Educ Couns. 2020;103:642–51. doi: 10.1016/j.pec.2019.09.022

18. van Veghel D, Daeter EJ, Bax M, Amoroso G, Blaauw Y, Camaro C, et al. Organization of outcome-based quality improvement in Dutch heart centres. Eur Hear J - Qual Care Clin Outcomes. 2019;6(1):49–54. doi: 10.1093/ehjqcco/qcz021

19. Stammen LA, Driessen EW, Notermans CCVI, Scheele F, Stassen LPS, Stalmeijer RE. How Do Attending Physicians Prepare Residents to Deliver High-Value, Cost-Conscious Care? Acad Med. 2019;95:764–70. doi: 10.1097/acm.0000000000003051

20. Nilsson K, Sandoff M. Leading implementation of the management innovation value-based healthcare at a Swedish University Hospital. J Hosp Adm. 2017;6(1):51–9. doi: 10.5430/jha.v6n1p51

21. Brandt Vegas D, Levinson W, Norman G, Monteiro S, You JJ. Readiness of hospital-based internists to embrace and discuss high-value care with patients and family members: a single-centre cross-sectional survey study. C Open. 2015;3(4):E382–6. doi: 10.9778/cmajo.20150024

22. Ryskina KL, Holmboe ES, Shea JA, Kim E, Long JA. Physician Experiences With High Value Care in Internal Medicine Residency: Mixed-Methods Study of 2003–2013 Residency Graduates. Teach Learn Med. 2018;30(1):57–66. doi: 10.1080/10401334.2017.1335207

23. Varley AL, Kripalani S, Spain T, Mixon AS, Acord E, Rothman R, et al. Understanding Factors Influencing Quality Improvement Capacity Among Ambulatory Care Practices Across the MidSouth Region: An Exploratory Qualitative Study. Qual Manag Health Care. 2020;29(3):136–41. doi: 10.1097/qmh.0000000000000255

24. Steinmann G, Van De Bovenkamp H, De Bont A, Delnoij D. Redefining value: a discourse analysis on value-based health care. BMC Health Serv Res. 2020;20(862):1–13. doi: 10.1186/s12913-020-05614-7

25. Busari JO, Duits AJ. The strategic role of competency based medical education in health care reform: a case report from a small scale, resource limited, Caribbean setting. BMC Res Notes. 2015;8(13):1–8. doi: 10.1186/s13104-014-0963-1

26. Nilsson K, Bååthe F, Erichsen Andersson A, Sandoff M. Value-based healthcare as a trigger for improvement initiatives. Leadersh Heal Serv. 2017;30(4):364–77. doi: 10.1108/LHS-09-2016-0045

27. Larsen KN, Kristensen SR, Søgaard R. Autonomy to health care professionals as a vehicle for value-based health care? Results of a quasi-experiment in hospital governance. Soc Sci Med. 2018;196:37–46. doi: 10.1016/j.socscimed.2017.11.009

28. Artenstein AW, Higgins TL, Seiler A, Meyer D, Knee AB, Boynton G, et al. Promoting high value inpatient care via a coaching model of structured, interdisciplinary team rounds. Br J Hosp Med. 2015;76(1):41–5. doi: 10.12968/hmed.2015.76.1.41

29. Beck JB, McDaniel CE, Bradford MC, Brock D, Sy CD, Chen T, et al. Prospective observational study on high-value care topics discussed on multidisciplinary rounds. Hosp Pediatr. 2018;8(3):119–26. doi: 10.1542/hpeds.2017-0183

30. Blayney DW, Simon MK, Podtschaske B, Ramsey S, Shyu M, Lindquist C, et al. Critical lessons from high-value oncology practices. JAMA Oncol. 2017;4(2):164–71. doi: 10.1001/jamaoncol.2017.3803

31. Demiris G, Hodgson NA, Sefcik JS, Travers JL, McPhillips M V, Naylor MD. High-value care for older adults with complex care needs: Leveraging nurses as innovators. Nurs Outlook. 2020;68:26–32

32. Robinson C, Lee JW, Davis KN, O’Connor M. Findings From FMAHealth’s Bright Spots in Practice Transformation Project. Fam Med. 2019;51(2):137–42. doi: 10.22454/FamMed.2019.163860

33. Hanak MA, McDevitt C, Dunham DP. Perceptions of Ambulatory Workflow Changes in an Academic Primary Care Setting. Health Care Manag (Frederick). 2017;36(3):261–6. doi: 10.1097/hcm.0000000000000174

34. Boynes S, Nelson J, Diep V, Kanan C, Pedersen DN, Brown C, et al. Understanding value in oral health: the oral health value-based care symposium. J Public Health Dent. 2020;80:S27–34. doi: 10.1111/jphd.12402

35. Cornell T. Leadership skills essential in the value-based care era. Leadersh Heal SERV. 2020;33:307–23. doi: 10.1108/LHS-12-2019-0079

36. Sondheim SE, Patel DM, Chin N, Barwis K, Werner J, Barclay A, et al. Governance Practices in an Era of Healthcare Transformation: Achieving a Successful Turnaround. J Healthc Manag. 2017;62(5):316–26. doi: 10.1097/jhm-d-15-00036

37. Gupta R, Steers N, Moriates C, Ong M. Association between hospitalist productivity payments and high-value care culture. J Hosp Med. 2018;13:16–21. doi: 10.12788/jhm.3084

38. Pereira V, Gabriel MH, Unruh L. Multiyear Performance Trends Analysis of Primary Care Practices Demonstrating Patient-Centered Medical Home Transformation: An Observation of Quality Improvement Indicators among Outpatient Clinics. Am J Med Qual. 2019;34(2):109–18. doi: 10.1177/1062860618792301

39. Grosso MJ, Courtney PM, Kerr JM, Della Valle CJ, Huddleston JI. Surgeons’ Preoperative Work Burden Has Increased Before Total Joint Arthroplasty: A Survey of AAHKS Members. J Arthroplasty. 2020;1–5. doi: 10.1016/j.arth.2020.01.079

40. O’Hara K, Tanverdi M, Reich J, Scudamore DD, Tyler A, Bakel LA. Qualitative Study to Understand Pediatric Hospitalists and Emergency Medicine Physicians’ Perspectives of Clinical Pathways. Pediatr Qual Saf. 2020;5:e270

41. Leep Hunderfund AN, Starr SR, Dyrbye LN, Baxley EG, Gonzalo JD, Miller BM, et al. Imprinting on Clinical Rotations: Multisite Survey of High- and Low-Value Medical Student Behaviors and Relationship with Healthcare Intensity. J Gen Intern Med. 2019;34:1131–8. doi: 10.1007/s11606-019-04828-8

42. Ryskina KL, Smith CD, Weissman A, Post J, Dine CJ, Bollmann KL, et al. U.S. Internal Medicine Residents’ Knowledge and Practice of High-Value Care: A National Survey. Acad Med. 2015;90(10):1373–9. doi: 10.1097/ACM.0000000000000791

43. Dainty KN, Golden BR, Hannam R, Webster F, Browne G, Mittmann N, et al. A realist evaluation of value-based care delivery in home care: The influence of actors, autonomy and accountability. Soc Sci Med. 2018;206:100–9. doi: 10.1016/j.socscimed.2018.04.006

44. Nilsson J, Mischo-Kelling M, Thiekoetter A, Deufert D, Mendes AC, Fernandes A, et al. Nurse professional competence (NPC) assessed among newly graduated nurses in higher educational institutions in Europe. Nord J Nurs Res. 2019;39(3):159–67. doi: 10.1177/2057158519845321

45. Meyer M. Qualifications and Competencies for Population Health Management Positions: A Content Analysis of Job Postings. Popul Heal Manag Heath Manag. 2017;0(0):475–85. doi: 10.1089/pop.2016.0197
